# Patterns of Recombination Activity on Mouse Chromosome 11 Revealed by High Resolution Mapping

**DOI:** 10.1371/journal.pone.0015340

**Published:** 2010-12-08

**Authors:** Timothy Billings, Evelyn E. Sargent, Jin P. Szatkiewicz, Nicole Leahy, Il-Youp Kwak, Nazira Bektassova, Michael Walker, Terry Hassold, Joel H. Graber, Karl W. Broman, Petko M. Petkov

**Affiliations:** 1 Center for Genome Dynamics, The Jackson Laboratory, Bar Harbor, Maine, United States of America; 2 Department of Statistics, University of Wisconsin, Madison, Wisconsin, United States of America; 3 Center for Reproductive Biology, Washington State University, Pullman, Wisconsin, United States of America; 4 Department of Biostatistics & Medical Informatics, University of Wisconsin, Madison, Wisconsin, United States of America; University of Minnesota, United States of America

## Abstract

The success of high resolution genetic mapping of disease predisposition and quantitative trait loci in humans and experimental animals depends on the positions of key crossover events around the gene of interest. In mammals, the majority of recombination occurs at highly delimited 1–2 kb long sites known as recombination hotspots, whose locations and activities are distributed unevenly along the chromosomes and are tightly regulated in a sex specific manner. The factors determining the location of hotspots started to emerge with the finding of PRDM9 as a major hotspot regulator in mammals, however, additional factors modulating hotspot activity and sex specificity are yet to be defined. To address this limitation, we have collected and mapped the locations of 4829 crossover events occurring on mouse chromosome 11 in 5858 meioses of male and female reciprocal F1 hybrids of C57BL/6J and CAST/EiJ mice. This chromosome was chosen for its medium size and high gene density and provided a comparison with our previous analysis of recombination on the longest mouse chromosome 1. Crossovers were mapped to an average resolution of 127 kb, and thirteen hotspots were mapped to <8 kb. Most crossovers occurred in a small number of the most active hotspots. Females had higher recombination rate than males as a consequence of differences in crossover interference and regional variation of sex specific rates along the chromosome. Comparison with chromosome 1 showed that recombination events tend to be positioned in similar fashion along the centromere-telomere axis but independently of the local gene density. It appears that mammalian recombination is regulated on at least three levels, chromosome-wide, regional, and at individual hotspots, and these regulation levels are influenced by sex and genetic background but not by gene content.

## Introduction

Identification of genes responsible for phenotypic traits is facilitated by linkage studies, which map their locations on chromosomes by genetic recombination analysis. This has been classically true since the first genetic maps were created [1], and has become increasingly important in contemporary efforts to identify genetic factors underlying disease predisposition in humans and experimental animals. The success of these studies ultimately depends on the locations of the crossovers separating a gene of interest from its adjacent genes, and this task is complicated by the fact that in many organisms, including humans and mice, recombination is not randomly distributed along the chromosomes. In mammals, the great majority of recombination events, perhaps all, are clustered in 1–2 kb genomic regions termed hotspots, which are typically separated from their neighboring hotspots by genomic distances from tens of kilobases to even megabases in length [2]–[4]. The hotspots themselves are not randomly positioned along the chromosomes but are often clustered in so-called “torrid zones” [5], or may be nearly absent from long genomic regions. Hotspot activities vary over several orders of magnitude when measured in sperm samples, from as high as 2–3 cM [2], [6] to less than 0.001 cM [6].

The recombination process begins in the leptotene stage of meiosis I by initiation of double-strand breaks catalyzed by the topoisomerase SPO11; these are eventually processed by two alternative pathways into crossovers and non-crossovers. The first of these pathways, known as double-strand break repair (DSBR), yields predominantly crossovers whereas the second, sequence-dependent strand annealing (SDSA), yields predominantly noncrossovers [7], [8]. Noncrossovers are recognized as gene conversions where a short segment of DNA in the initiating chromatid acquires the sequence of its recombination partner. Positioning of the double-strand breaks is genetically regulated by trans-acting factors in yeast [9], [10] and in mammals [11], [12] acting through posttranslational modification of histones at hotspot sites [13]–[17]. Recently, PRDM9 was identified as the major factor regulating hotspot activity in mice and humans [18]–[20].

In most organisms the number of crossover events on each bivalent is tightly regulated. At least one crossover per chromosomal arm is required for successful meiosis in most organisms, as observed by both genetic and cytological studies [21]–[23], whereas in humans the rule appears to be one crossover per chromosome [24]. Part of this control involves a choice as to which of the many DSBs will become crossovers. In mice, about 250–400 double-strand breaks are initiated, but only about one-tenth of them are processed into crossovers [25]–[27]. The number of crossovers in budding yeast and possibly in higher eukaryotes in general is regulated by a still enigmatic mechanism imposing crossover homeostasis [28], which ensures that a relatively constant number of the highly variable number of initial DSBs is processed into crossovers. In part the total number of crossovers on each chromosome is restricted by crossover interference, which prevents crossovers from occurring near each other and is very strong in mammals, operating over distances spanning tens of megabases [29], [30]. The opposing requirements of having at least one crossover per chromosome, but limiting their density by interference, results in a strong tendency for shorter chromosomes to have more crossovers per unit length than larger chromosomes [31].

In many organisms of various taxonomic groups, genetic maps have different length in the two sexes (for review, see [32]). The female genetic map is 1.6 times longer than the male map in humans [33], [34] and 1.09 times in mice [35]. The main reason for this difference is crossover interference, which operates over shorter genomic distances in females than in males and is related to synaptonemal complex length at the pachytene stage of meiosis I [30], [36]. In addition to differences in the intensity of interference, the positioning of recombination activity along the chromosomes differs significantly between the two sexes. Male recombination rates tend to be higher close to telomeres whereas female recombination is more evenly distributed [23], [37]. Sex specificity has also been detected at the level of individual hotspots [38].

Recently, we presented a detailed study of the location and relative activity of recombination hotspots on mouse chromosome 1 in a cross involving two inbred strains, C57BL/6J and CAST/EiJ [2]. Two fundamental questions raised by this work are whether our observations are specific to a particular chromosome, or apply more generally across the genome, and to what extent they are influenced by the length of the chromosome and its gene content. To address these questions, we present in this study our analysis of high resolution mapping of recombination on mouse chromosome 11, which is a medium-size but the most gene-dense chromosome in the mouse genome, keeping the genetic background of the analysis the same, a C57BL/6J x CAST/EiJ cross. Thirteen new hotspots were mapped to <8-kb resolution providing material for further research.

## Results

### High Resolution Mapping

We studied recombination rates along the entirety of mouse chromosome 11 in the meioses of C57BL/6J (B6) and CAST/EiJ (CAST) F1 hybrids of both sexes at an average resolution of 127 kb. To test for potential effects parental imprinting might have on recombination, the F1 animals were produced by reciprocal crosses, and then backcrossed to C57BL/6J. Mapping the location of crossovers in these backcross progeny provided information on the recombination events arising in the F1 hybrids. A total of 5858 progeny were genotyped, of which 1465 were offspring of female B6xCAST, 1537 of female CASTxB6, 1343 of male B6xCAST, and 1513 of male CASTxB6. Backcross offspring were genotyped in four consecutive rounds with single nucleotide polymorphism (SNP) assays developed using competitive allele specific PCR (KASPar, www.kbioscience.co.uk) and Amplifluor system [39] (see Materials and Methods). In the first round, all progeny DNAs were mapped over the entire chromosome at 15-Mb resolution. This was sufficient to detect virtually all crossovers, given the strong interference in mouse meiosis [29]. In the second round, the crossovers occurring in each interval were mapped using additional SNP markers to an average physical resolution of 225 Kb. In the third round, all regions showing recombination rates higher than 0.5 cM in either female or male meiosis were genotyped to ∼50 kb resolution. In the fourth round, the regions retaining recombination rates of 0.5 cM or higher were mapped down to hotspot resolution. In each round, flanking markers were typed to confirm the presence of a valid crossover, ensuring an extremely low error rate. In all, we detected and localized 4829 crossover events on chromosome 11, reaching a genetic resolution of 0.017 cM in the combined offspring.

Among the crossovers occurring along the chromosome, 97.0% were mapped to <300 kb resolution. Among these, 3.3% were mapped to fewer than 8 kb, 30.8% to 8–50 kb, 26.2% to 50–100 kb, 25.2% to 100–200 kb and 14.5% to 200–300 kb resolution. All markers used in this study, their positions according to NCBI Build 37, physical resolution and the number of crossovers in each interval are included in Table S1. Thirteen hotspots with activities of 0.7 cM or higher in either female or male meiosis are shown in Table 1.

**Table 1:** Individual hotspots showing activity of 0.7 cM or higher in female or male meiosis.

			Hotspot Activity (cM)	
Name	Position of the centromere-proximal marker (Mb, B37)	Hotspot Size (kb)	Female	Male
Egfr-1	16.748792	7.212	0.10	0.04
Egfr-2	16.756004	1.824	0.10	0.04
Egfr-3	16.763416	3.313	0.50	0.25
Peli1-1	20.779098	0.509	0.00	0.07
Peli1-2	20.790039	6.308	0.03	0.28
Tekt3-1	62.88897	3.089	0.00	0.07
Tekt3-2	62.892059	3.213	0.10	1.23
Ankfn1	89.470824	4.518	1.27	1.30
Tmem106a	101.45505	1.536	0.17	0.25
1700012B07Rik	109.645559	6.291	0.00	0.11
GP112-1*	112.206482	1.755	0.10	0.07
GP112-2*	112.273362	3.862	0.50	0.21
Slc9a3r1	115.039527	1.983	0.03	0.25

Hotspot names match the name of the closest gene.

*Hotspot in a gene-poor region.

### Regional Variation

The sex-averaged genetic map length of chromosome 11 in the B6xCAST cross was 82.8 cM, which represents an average rate of 0.70 cM/Mb across 118.4 Mb, excluding the centromere adjacent 3.1 Mb for which no sequence information is available according to NCBI sequence build 37. This rate is higher than the genome wide average of 0.56 cM/Mb [40], and the genetic length of Chromosome 11 in this cross is very close to the length of Chromosome11 from the integrated map (83.1 cM) reported in Mouse Genome Database [41] and from the recently published revised map (80.85 cM) [35].

**Figure 1:**
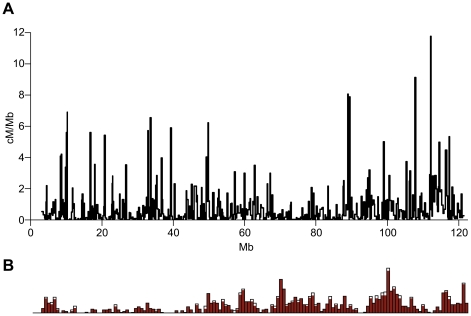
Recombination patterns on mouse chromosome 11. **A. Sex-averaged recombination map of chromosome 11**. Recombination events were mapped to an average resolution of 225 Kb in two consecutive rounds. Recombination rates are presented as cM/Mb to adjust for interval size. **B. Gene density on chromosome 11 (from ENSEMBL,** www.ensembl.org **)**. The X-axis is to scale with 1A. The height of the peaks represents the relative gene density in 1-Mb intervals.

Recombination activity was distributed very unevenly along the chromosome (Fig. 1). At 225 kb resolution, recombination activity was found in only 71.6% of all intervals along the chromosome, the remaining 28.4% being completely devoid of recombination. Regionally higher recombination rates were prominent at 8–10 Mb, 32–40 Mb, 45–51 Mb, 87–90 Mb and 110–117 Mb. At this resolution, there were 14 intervals with recombination rates above 5 cM/Mb, all clustered in either the centromeric third (10–50 Mb) or telomeric third of the chromosome (88–117 Mb). Generally, recombinationally active regions were separated by regions having significantly lower recombination rates. There were several regions of one megabase or more in length that lacked recombination; these were most abundant in the central region of the chromosome between 50–88 Mb.

### Sex specificity

We found 2537 recombination events in 3002 female meioses and 2292 recombination events in 2856 male meioses. The total lengths of female and male recombination maps of Chromosome 11 were 84.8 cM and 80.9 cM, which account for average rates of 0.716 and 0.683 cM/Mb, respectively, and a female: male ratio of 1.05. This length difference was statistically significant (p = 0.018 by χ^2^ test) and was manifested by the presence of more multiple crossovers in female compared to male meiosis (p = 10^−4^ by χ^2^ test). In total, we found 529 double crossovers, 10 triple crossovers and 1 quadruple crossover in the progeny of female hybrids, compared to 398 double and one triple crossover in the progeny of male hybrids. The frequency with which chromosomes with different numbers of crossovers were observed is summarized in Table 2.

**Table 2:** Frequencies of offspring with different number of crossovers on chromosome 11.

		Number of Crossovers per Chromosome					
		0	1	2	3	4	Total
Female	Number	973	1489	529	10	1	3002
	Frequency	0.324	0.496	0.176	0.003	0.0003	
Male	Number	961	1496	398	1	0	2856
	Frequency	0.336	0.524	0.139	0.0004	0	

**Figure 2:**
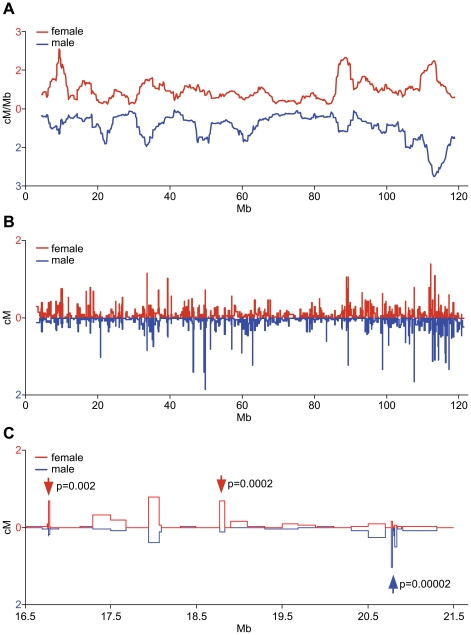
Sex-specific recombination map of chromosome 11. **A.** Sex-specific recombination rates averaged over 2-Mb sliding window. **B.** Fine mapping of recombination activity. The entire chromosome was mapped at 225 Kb resolution and then highly active intervals showing sex-specific rates were mapped further to <50 kb resolution. The recombination rates are expressed as cM to account for these resolution differences. Female, red line above, male, blue line below. **C.** Expanded view of the region between 16.5-21.5 Mb. Hotspots with higher activity in female (red arrows) or male meiosis (a cluster marked with blue arrow) are shown. The p-values of the difference were determined by Fisher's exact test.

The distribution of recombination events along the chromosome was also significantly different between the sexes (p<10^−4^ by randomization test) (Fig. 2A). A total of 28 intervals showed sex-specific recombination rates by Fisher's Exact Test after correction for multiple testing (q<0.05) (Fig. 2B and Table S2). Among the 71.6% of intervals showing any recombination, slightly more than half (58.8%) were active in both sexes, about a quarter (28.4%) were only active in females and one-eighth (12.8%) were only active in males. There was also a marked sex difference in the extent to which recombination tended to be concentrated in highly active regions; 38% of all male activity occurred in intervals with recombination rates above 0.5 cM, but only 26% of all activity in females occurred in intervals with similar rates. Conversely, intervals with lower recombination rates, between 0.2–0.5 cM, contained 40% of female activity and 36% of male activity. These differences were also reflected in the location of recombination along the chromosome. Female recombination rates were substantially higher in the pericentromeric 19 Mb and in the regions between 25–45 Mb and 64–90 Mb, whereas male recombination rates were higher in the telomeric 10 Mb and in the region between 45–63 Mb (Fig. 2A). Significant differences in recombination rates were also observed at the level of individual hotspots (Fig. 2C).

### Parent of Origin Effects

The distribution of recombination rates over the entire chromosome showed a statistically significant difference between the two parental directions (B6xCAST and CASTxB6) in females (p = 0.04) but not in males (p = 0.15). Combined sex-averaged data showed more pronounced difference between the two directions (p = 0.0026). No single interval showed statistically significant difference between the two directions in either females or males after correction for multiple testing.

### Interference

**Figure 3:**
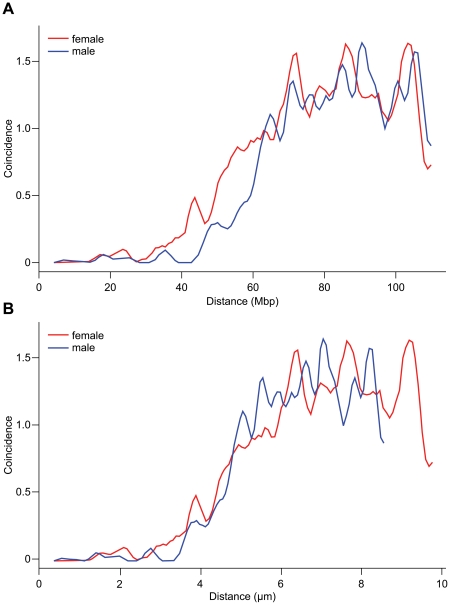
Sex Specificity of Interference. **A.** Coefficient of coincidence (Z) as a function of intercrossover distance in Mb. **B.** Coefficient of coincidence (Z) as a function of intercrossover distance in µm of synaptonemal length at the pachytene stage of meiosis I. Female, red line; male, blue line.

Interference operated on significantly shorter distances in females than in males as measured by the coefficient of coincidence (Z) [42] (Fig. 3A). In female meiosis, interference was nearly complete (Z<0.1) up to 28 Mb and then faded away between 28 Mb and 57 Mb, with Z = 0.5 at 44 Mb. In males, nearly complete interference was found up to 42 Mb and then it decreased between 42 Mb and 80 Mb, with Z = 0.5 at 60 Mb. These differences in interference parameters explain why triple recombinants are found almost exclusively in females – the size of chromosome 11 (121 Mb) provides enough space for three events in females but is barely sufficient in males.

In our previous work [30] we found that the difference in interference between the two sexes on large chromosomes is related to the length of synaptonemal complexes at the pachytene stage of meiosis [36]. To test whether this effect holds in medium-size chromosomes, we measured synaptonemal complex length of chromosome 11 at pachytene in 74 oocytes and 57 spermatocytes of 5 female and 5 male C57BL/6J mice stained with antibodies against SYCP3 (a component of the axial/lateral elements of the synaptonemal complex) [27] and a chromosome 11-specific DNA probe. The average lengths of chromosome 11 synaptonemal complexes were 10.8±1.7 (SD) µm in females and 9.5±2.0 (SD) µm in males. The difference between female and male synaptonemal complex lengths was statistically significant by two-way *t*-test (p<0.0001). Plotting interference as a function of synaptonemal complex length revealed the same relation on chromosome 11 as on chromosome 1 – the coincidence curves overlapped, with Z = 0.5 at 4.5 µm suggesting that interference acts similarly in males and females on the micron scale (Fig. 3B).

### Recombination and gene density

Overall, we did not detect a significant correlation between recombination and gene density. Over distances up to 800 kb, there was no statistically significant effect. Over 800 kb, there was statistically significant correlation between male recombination rates and gene density but this effect disappeared at distances over 1 Mb. (Table S3). The lack of correlation over the entire chromosome was due to opposing regional trends, with a significant positive correlation between gene density and recombination in either sex in the centromere-proximal half and the telomere-proximal 20 Mb but negative correlation in the region between 60–95 Mb (Fig. 1A and 1B, Table S4). A slightly negative correlation between exons and female recombination rates was found (p<0.05) as well as between transcription start sites and female recombination (p<0.05). No significant correlation between these features and male recombination rates was found.

### Recombination and sequence features

Across the chromosome, six genomic features (DNA transposons, LINE, LTR, low complexity (poly-purine/poly-pyrimidine stretches) and simple repetitive elements, GC content) were found to have significant correlations in four or more of the nine window sizes (200 kb to 1000 kb, 100 kb increment per window size) (Tables S3 and S4). GC content, DNA repetitive elements, LINE repetitive elements, and low complexity repetitive elements showed significant positive correlation in all window sizes in at least one sex. DNA and simple repetitive elements were most significant across all crosses and window sizes in both sexes. In females, recombination rate was negatively correlated with LTR and positively correlated with low complexity repetitive elements. In males, recombination rate was negatively correlated with LINE and positively correlated with GC content and low complexity repetitive elements.

PRDM9, a zinc finger protein with a histone H3 lysine-4 trimethylation activity, was recently identified as a gene determining hotspot positioning [18], [19], with the two strains used in this study having different alleles [18]. These alleles should bind different DNA sequences [19] based on a zinc finger DNA-binding predictions [43]. We reasoned that if PRDM9 regulates the activity of a significant proportion of hotspots, it should be possible to find a correlation between the predicted binding sequences and recombination rates. At the resolution achieved chromosome-wide, we did not find a statistically significant correlation between the predicted binding sequences of the two alleles and recombination rates (Table S5).

## Discussion

Our detailed analysis of recombination along mouse chromosome 11 provides an opportunity to determine the generality of recombination anatomy features and to test the effects of chromosome length while keeping the genetic resolution and genetic background constant [2].

The distribution of recombination events on chromosomes 1 and 11 follows a similar regional pattern. Regional peaks of higher- and lower-than average recombination activity spanning 5–10 Mb in size are present in the centromere-proximal half of the chromosome, followed by a substantially longer region of low recombination (30–40 Mb in size) in the middle third of the chromosome and generally elevated recombination rates in the telomere-proximal one-third to one-fourth of the chromosome. The recombination rates on most mouse chromosomes except the shortest chromosome 19 [35] and on the long arms of human chromosomes [44] follow similar pattern. This general trend suggests a common principle of chromosome-wide distribution of recombination influenced by the centromere-telomere axis and can be considered as a separate, chromosome-wide level of control of recombination activity.

Our present results provide additional evidence for at least two other levels of control that regulate positioning and activity of crossover events, confirming prior results in mice [2] and humans [45], [46], one at a megabase-scale which we consider a regional level of control, and another at the positioning of individual hotspots which we consider a local level of control. The regional distribution is determined by the positioning of the crossover relative to the centromere-telomere axis and the interference, with both of them showing sex specificity. However, the mechanisms that impose this regional control are largely unknown. It is tempting to think that regional variation in chromatin structure creating more open or more closed chromatin is involved, as shown in yeasts [47], and also suggested by the differences of hotspot distribution in humans (reviewed in [48]) and mice [2], [49]. However, we did not detect any correlation between recombination activity and some of the predictors of more open chromatin structure such as exon density and transcription start sites. At a local level, there is an emerging body of data which sheds light on the regulation of individual hotspot activity as determined by the interaction of their own sequences with the products of trans-acting genes such as PRDM9 [12], [50].

Sex specific effects follow similar patterns on chromosomes 1 and 11. Female recombination is more evenly distributed along the chromosomes and is present in larger numbers of intervals at ∼200 Kb resolution. The somewhat lower overall level of male recombination is concentrated in fewer intervals, albeit with higher activity; a substantial part of it is present at the 10–15 Mb near the telomeres, and is relatively low near the centromeres. At the local level, females and males tend to use similar sets of hotspots, but nearly half of all intervals exhibiting any activity were active in only one sex. Despite this, hotspots showing higher activity in one sex may be found in regions where the opposite sex generally shows a higher regional rate. In a 25-Mb interval on chromosome 1, where we reached almost entirely hotspot resolution (<8 kb), we estimated that females use more hotspots than males, although with lower activity per hotspots; the data presented here hints that this must also be true for chromosome 11 because we found recombination in more intervals in females than in males. Coop et al. [51] have reached similar conclusions about sex specificity of hotspot usage in humans. Crossover interference can explain to a great extent the sex-specific differences in total recombination rates along the chromosomes. Interference operates over shorter genomic intervals in females than in males and allows placement of more multiple crossovers in female meiosis; it is related to the length of synaptonemal complexes which are longer in females than in males. This principle essentially applies in similar fashion for chromosomes 1 and 11. When measured in megabases of genomic lengths, the interference distances are significantly longer on chromosome 1 than chromosome 11 (in females Z = 0.5 at 60 Mb on chromosome 1 vs.44 Mb on chromosome 11, ratio of 1.36; in males Z = 0.5 at 95 Mb vs. 60 Mb, ratio of 1.53). When they are measured in microns of synaptonemal complex length, the interference distances on both chromosomes are not much different (Z = 0.5 at 4.46 µm for chromosome 1 vs. 3.94 µm for chromosome 11 in females, ratio of 1.13; Z = 0.5 at 5.85 µm vs. 4.88 µm in males, ratio of 1.20).

Interference might also be involved in the presence of long regions with low recombination activity in the middle third of each chromosome. In male meiosis, the significantly higher recombination rate near the telomeres (24% of the male recombination activity is located in the telomere-proximal 9 Mb compared to 13% in females) determines to a great extent the positioning of the second crossover at distances near to or exceeding the interference distance, which is where the second peak of male recombination activity is located. This raises the interesting possibility that the decision to process double-stranded breaks into crossovers rather than non-crossovers may be implemented consecutively in a wave along the chromosome with the placement of the first crossover occurring near the telomere, where recombination rates are concentrated over a short span, followed by placement of a possible second crossover at a distance according to the rules of interference. It is not as clear whether this rule also applies to female rates. Further investigation of temporal placement of crossover events is needed to examine this hypothesis in detail.

We found statistical evidence for possible involvement of imprinting in determining recombination activity over the entire chromosomes for both chromosomes 1 and 11, although not at the level of individual hotspots. Combining the data for both chromosomes confirmed this conclusion with increased statistical significance (p = 0.003 and 0.01 for female and male reciprocal crosses, respectively). However, the imprinting effect at any single hotspot is only quantitative; we did not find hotspots active in one parental direction but not the reciprocal. Examples of imprinting at individual hotspots have been found on mouse chromosome 7 [52]. Further detailed investigation of hotspot activity in sperm of reciprocal F1 animals may provide the necessary evidence for imprinting at the level of individual hotspots.

We found significant correlations of recombination rates with GC content, DNA repetitive elements, LINE repetitive elements, and low complexity repetitive elements but not with gene density, transcription start sites and exons. It is possible that sequence elements controlling recombination hotspot activity may be more active when embedded in repetitive elements as is the case in humans [46], [53]. We did not find significant correlation between recombination activity and predicted DNA binding motifs of PRDM9 alleles. This lack of correlation, however, may not be surprising. First, the rules determining binding of Zn fingers to DNA motifs are inferred from proteins with 1–3 Zn fingers and may be different for multiple, tandemly arrayed Zn fingers [43]. Second, it is possible that the presence of such DNA motifs is necessary but not sufficient condition for hotspot activity, which may depend on the general context of chromatin structure over longer intervals. Finally, the motifs determine hotspot activation at the initiation of recombination events [12]; since interference imposes significant constraints on crossover positioning, the set of crossover hotspots could be only a fraction of the initiating hotspots, or show different frequency distribution along the chromosomes.

Our present data confirm that the same general principles underlie the recombination landscapes of two different mouse chromosomes with the centromere-telomere axis of the chromosome and interference being the main factors regulating recombination on a regional level. Genetic background and sex determine to a great extent the actual placement and activity of recombination hotspots, resulting in at least three levels of recombination control – on entire chromosomes, on megabase scale, and at the level of hotspots. An open question is how local and distal factors shaping recombination rates, such as trans-acting DNA-binding proteins, chromatin structure, and interference, combine their effects to achieve similar regional recombination rates along the chromosomes when using different sets of hotspots.

## Materials and Methods

### Ethics Statement

All animal experiments were approved by the Animal Care and Use Committee of The Jackson Laboratory (Animal Use Summary #04008).

### Strains and crosses

C57BL/6J and CAST/EiJ were obtained from The Jackson Laboratory, Bar Harbor, USA. F1 hybrids were produced by reciprocal crosses in which either strain was the female or male parent. These hybrids were then backcrossed to C57BL/6J and recombination was detected in their progeny. All parents and F1 hybrids were genotyped for three markers on each chromosome to ensure strain identity using DNA isolated from tail tips.

### Genotyping and data cleaning

DNA for genotyping was prepared from spleens of weaned progeny as described before [2]. All progeny were genotyped at 10 Mb resolution using previously described assays [54] for single nucleotide polymorphisms (SNPs) based on Amplifluor technology [39]. Individuals with a gap of >35 Mb between typed markers were omitted from subsequent analyses. Recombination was detected as a transition from homozygous to heterozygous genotype or vice versa. New Amplifluor assays were developed for the subsequent rounds of genotyping using the publicly available SNP database of the Mouse Phenome Project (http://phenome.jax.org/pub-cgi/phenome/mpdcgi?rtn=snps/door). All recombinants detected in the first round of genotyping were subsequently mapped to increased resolution until reaching the maximum hotspot resolution. In each round, the flanking markers from the previous round were retyped to confirm the validity of the recombinants. A total of 238,791 genotypes were produced. Of them, 11,696 did not amplify, 16,116 were inconclusive, and 104 were contradicting calls when retyped. These 27,916 genotypes were excluded from the analysis. A list of all markers used in this study is available as part of the Online Supporting Material (Table S1). The positions of all markers are in accordance with NCBI Build 37.

### Cytological analyses

Slides were immunostained using similar methodology to that of Anderson et al.[55], using a primary antibody against SYCP3 (Santa Cruz Biotechnology) to detect pachytene stage synaptonemal complexes. Using a Zeiss epifluorescence microscope, pachytene cells were identified and their locations recorded for subsequent fluorescence in situ hybridization (FISH) analyses.

Immunostained slides were then denatured and a FISH probe specific for chromosome 11 (StarFish whole chromosome paint probe; Cambio) was applied to the slides to identify chromosome 11. Pachytene stage cells were re-located and the length of the chromosome 11 synaptonemal complexes determined using a Zeiss Axiovision measuring tool.

### Statistical analysis

All the analyses were performed using R (http://www.r-project.org) on the untransformed data (i.e. numbers of crossovers per interval) as previously described [2]. The calculated raw *P*-values were then transformed into *q*-values based on Storey and Tibshirani [56]. A *q*-value cutoff of 0.1 (equivalent to a false discovery rate (FDR) of 10%) was used to determine significant intervals.

For estimating the coincidence function, we considered pairs of intervals, I_1 and I_2, of width 3 Mb, whose midpoints were separated by a fixed distance, and calculated average proportion (XOs in I_1 and I_2)/{average [Proportion (XO in I_1) x Proportion (XO in I_2)]}. The averages in the numerator and denominator are overall all such pairs of intervals.

### Correlation between genomic features and recombination

The exon and transcript data was downloaded from the UCSC MySQL server (http://genome.ucsc.edu/FAQ/FAQdownloads#download29) using data from NCBI Build 37 of the mouse genome. The density is the fraction of the genome within transcribed sequences or exon coding regions, respectively, calculated in 50 Kbp blocks. Transcription start site density represented the number of 5′-gene ends per 50 kb. For exon and transcript coverage, overlapping between genes on both strands was treated as a continuous exon or transcript. Transcriptional starts only considered unique start sites; i.e., if two or more transcripts had a common start site, the site was only counted once.

The recombination rate for each window was computed as the sum of the recombination rates of the observed regions. When a region fell across multiple windows, each window received a fraction of the recombination rate determined by the fraction of the region overlapping the window. The window metric for transcription start sites and the degenerate motif were the total of occurrence within the window. For all other features, the window metric was the proportion of the window comprised of the feature. In the event of an overlap, e.g., overlapping genes, each position was considered only once. Correlation was calculated using the Pearson's product-moment correlation between the normalized recombination rate (cM/Mb) and the genomic feature (i.e., gene density, exon density, transcription start sites). The significance of the correlation was determined by 1000 bootstrap iterations, counting the number of correlations with an absolute value greater than the absolute value of the original correlation. Repetition of the bootstrap analysis found the results to be robust and no significant improvement was observed when using more than 1000 iterations.

Nucleotide values for the DNA recognition sequence of PRDM9 alleles were obtained by extrapolating known C2H2 proteins to DNA mappings as compiled in the Zinc Finger Database [57] similar to methods of [19]. A position specific weight matrix was used to count occurrences across genome of the recognition patterns for both the B6 and CAST alleles [58].

## Supporting Information

Table S1 **Recombination data**. All markers are presented with their dbSNP rs-numbers, and placed in increasing order of their positions according to NCBI Build 37. The number of crossovers and total number of samples tested are presented for each interval between the marker on the same row and the next marker.
(PDF)

Table S2 **Intervals with sex-specific differences in recombination rates**. Female and male numbers of recombinants are presented together with p-values of the difference calculated by Fisher's exact test and q-values correcting for multiple testing (see Material and Methods). All intervals with q<0.1 are included.
(PDF)

Table S3 **Correlations between recombination rates and different genomic features at different window sizes**.
To identify genomic patterns associated with recombination hotspots, we tested the correlation between recombination rates and genomic features. In total, 19 features were examined: the GC content, gene density, exon density, number of transcription start sites, and 15 classes of repetitive elements.
In order to compare recombination rate with genomic features, the chromosome was divided into several adjacent, non-overlapping windows. The minimum size of the window was determined by the average interval between SNPs used to identify recombination hot spots. Exact points of recombination are not known. Rather, it is the *interval* between SNPs where recombination has taken place. Subdivision of these regions may result in a false positive. For example, if recombination events took place in a 100 kb region and this is divided in half, it is not known how many recombinations took place in either half. This will lead to false correlations for regions where recombination was falsely assumed.
The distribution of these intervals was bi-modal with peaks around 50 kb and 200 kb. These densities were the result of the hotspot detection process as successive round of assays focused on smaller regions. Minimum window size must be at least equal to the largest of these peaks to minimize disruption. Based on these results, we tested for correlation in window sizes of 200 kb to 1,000 kb in 100 kb increments for a total of nine window sizes. Correlations were also performed for window sizes from 50 kb to 100 kb in 10 kb increments. Focus of the analysis was on the 200 kb to 1,000 kb range. Windows were defined beginning at 3 Mb and ending at 121.7 Mb, spanning all markers used to detect recombination.
The recombination rate for each window was computed as the sum of the recombination rates of the observed regions. When a region fell across multiple windows, the each window received a fraction of the recombination rate determined by the fraction of the region overlapping the window. The window metric for transcription start sites and the degenerate motif were the total of occurrence within the window. For all other features, the window metric was the proportion of the window comprised of the feature. In the event of an overlap, e.g., overlapping genes, each position was considered only once.
Correlations were computed using the Pearson correlation using the cor.test command in R and C++ code. The significance level for the C++ code was determined using 100,000 permutations of the data.
(XLSX)

Table S4 **Correlations between recombination rates and different genomic features in 10-Mb segments along the chromosome**.
In addition to correlation across the entire chromosome 11, it is of interest to identify localized correlations between genomic features and recombination. The chromosome was divided into twelve sections of 10 Mb. The sectional analysis was performed twice for each section size category per window size. The first began at 3 Mb and proceeded through 123 Mb, which was truncated to 121.7 Mb. The second began at 121.7 Mb and continued to 1.7 Mb, truncated to 3 Mb. Correlations were calculated for the same genomic features as in Table S3.
(XLSX)

Table S5 **Correlation between recombination rates and inferred binding motifs of C57BL/6J and CAST/EiJ alleles of Prdm9**. Allele-specific Prdm9 binding motifs were as described in [19]:
C57BL/6J motif – GTnTCnTGnTGnTnnTnnnnnnTnnnnnnnTTnTG
CAST/EiJ motif – GTnnTnTnnTGnnTnnnnnTnTnnnTnTTnTG
Correlations and their significance were calculated for window sizes of 200 kb, 400 kb and 500 kb as described for Table S3.
(XLSX)
