# Supplementary material for: Patterns of Recombination Activity on Mouse Chromosome 11 Revealed by High Resolution Mapping
Source: PLoS One. 2010 Dec 8;5(12):e15340. doi: 10.1371/journal.pone.0015340 (PMC2999565; doi:10.1371/journal.pone.0015340)
Supplement: Table S1 — Recombination data. All markers are presented with their dbSNP rs-numbers, and placed in increasing order of their positions according to NCBI Build 37. The number of crossovers and total number of samples tested are presented for each interval between the marker on the same row and the next marker. (PDF) [file pone.0015340.s001.pdf]

Marker	Position (Mb, B37)	Distance (Mb)	Number of Recombinants Female (BxC)	Female(CxB)	Male (BxC)	Male (CxB)	Number of samples Female (BxC)	Female(CxB)	Male (BxC)	Male (CxB)
rs26884366	3.113808	0.372493	4	2	2	0	995	1014	822	931
rs26913823	3.486301	0.405202	2	1	2	0	995	1014	822	931
rs26884450	3.891503	0.198786	1	0	0	0	995	1014	822	931
rs26913550	4.090289	0.002198	0	0	0	0	995	1014	822	931
rs26913544	4.092487	0.174678	0	0	1	0	995	1014	822	931
rs26882867	4.267165	0.02609	0	0	0	0	995	1014	822	931
rs26882835	4.293255	0.007072	0	0	0	0	995	1014	822	931
rs26882819	4.300327	0.108406	4	1	0	0	995	1014	822	931
rs3659787	4.408733	0.171229	5	7	6	4	1465	1537	1343	1513
rs26922830	4.579962	0.109592	0	0	0	0	1465	1537	1343	1513
rs26901862	4.689554	0.15999	0	0	0	2	1465	1537	1343	1513
rs26885878	4.849544	0.242591	2	2	0	0	1465	1537	1343	1513
rs3165903	5.092135	0.199994	0	0	0	1	1465	1537	1343	1513
rs26885253	5.292129	0.200541	0	4	0	1	1465	1537	1343	1513
rs26885091	5.49267	0.19001	1	2	0	0	1465	1537	1343	1513
rs26931523	5.68268	0.00817	0	0	0	0	1465	1537	1343	1513
rs26931514	5.69085	0.22464	1	1	1	2	1465	1537	1343	1513
rs26914468	5.91549	0.210352	1	6	1	1	1465	1537	1343	1513
rs26899824	6.125842	0.164946	0	1	0	2	1465	1537	1343	1513
rs26885720	6.290788	0.215583	4	8	2	1	1465	1537	1343	1513
rs26911221	6.506371	0.227911	3	1	2	0	1465	1537	1343	1513
rs26895832	6.734282	0.134679	1	1	1	1	1465	1537	1343	1513
rs26880284	6.868961	0.218646	7	6	1	3	1465	1537	1343	1513
rs26897185	7.087607	0.17833	0	0	0	0	1465	1537	1343	1513
rs26881286	7.265937	0.265284	2	0	0	0	1465	1537	1343	1513
rs26910884	7.531221	0.165405	0	0	0	0	1465	1537	1343	1513
rs26896285	7.696626	0.204551	0	0	0	0	1465	1537	1343	1513
rs26881534	7.901177	0.188555	2	1	1	2	1465	1537	1343	1513
rs26909950	8.089732	0.262145	0	0	0	0	1465	1537	1343	1513
rs3685325	8.351877	0.05032	1	1	1	0	1465	1537	1343	1513
rs3088673	8.402197	0.087236	5	8	3	5	1465	1537	1343	1513
rs26895284	8.489433	0.09215	3	2	7	2	1465	1537	1343	1513
rs26881897	8.581583	0.079396	1	0	0	0	1465	1537	1343	1513
rs26881891	8.660979	0.028326	2	2	2	1	1465	1537	1343	1513
rs26881863	8.689305	0.202011	2	2	0	0	1465	1537	1343	1513
rs26894657	8.891316	0.195092	5	5	3	2	1465	1537	1343	1513
rs26911561	9.086408	0.21272	2	4	0	0	1465	1537	1343	1513
rs26896702	9.299128	0.180106	1	1	0	0	1465	1537	1343	1513
rs3703920	9.479234	0.015951	0	0	0	0	1465	1537	1343	1513
rs26927593	9.495185	0.180822	6	6	0	0	1465	1537	1343	1513
rs26896523	9.676007	0.046228	0	0	0	0	1465	1537	1343	1513
rs26896490	9.722235	0.056799	0	0	0	0	1465	1537	1343	1513
rs26880739	9.779034	0.133499	8	15	3	1	1465	1537	1343	1513
rs26880554	9.912533	0.005208	0	0	0	0	1465	1537	1343	1513

rs26880523	9.917741	0.174996	3	1	1	0	1465	1537	1343	1513
rs26895754	10.09274	0.090399	9	14	2	5	1465	1537	1343	1513
rs26895633	10.18314	0.043097	0	1	0	0	1465	1537	1343	1513
rs26895554	10.22623	0.043118	1	1	0	0	1465	1537	1343	1513
rs26895492	10.26935	0.03662	6	5	3	1	1465	1537	1343	1513
rs26881032	10.30597	0.174262	0	0	0	0	1465	1537	1343	1513
rs6375717	10.48023	0.206951	0	0	0	0	1465	1537	1343	1513
rs26909491	10.68718	0.198655	0	0	0	0	1465	1537	1343	1513
rs26894424	10.88584	0.206293	1	0	0	0	1465	1537	1343	1513
rs26878989	11.09213	0.193076	0	0	0	0	1465	1537	1343	1513
rs28220128	11.28521	0.200639	0	0	0	0	1465	1537	1343	1513
rs28205816	11.48585	0.199457	1	2	1	2	1465	1537	1343	1513
rs28185790	11.6853	0.040499	2	1	0	0	1465	1537	1343	1513
rs26855246	11.7258	0.029935	1	0	0	0	1465	1537	1343	1513
rs26855172	11.75574	0.05621	0	0	0	0	1465	1537	1343	1513
rs26855078	11.81195	0.051983	7	9	1	4	1465	1537	1343	1513
rs26855017	11.86393	0.03576	1	0	0	0	1465	1537	1343	1513
rs26839294	11.89969	0.212157	2	0	5	2	1465	1537	1343	1513
rs26832096	12.11185	0.179893	2	2	3	4	1465	1537	1343	1513
rs26860407	12.29174	0.206337	0	0	0	0	1465	1537	1343	1513
rs26834194	12.49808	0.196375	1	1	0	0	1465	1537	1343	1513
rs26858209	12.69445	0.195088	0	0	0	0	1465	1537	1343	1513
rs26842498	12.88954	0.206158	0	0	0	0	1465	1537	1343	1513
rs26828902	13.0957	0.180884	0	0	0	0	1465	1537	1343	1513
rs26861406	13.27658	0.196282	0	0	0	1	1465	1537	1343	1513
rs26833362	13.47287	0.413814	0	0	0	0	1465	1537	1343	1513
rs26863480	13.88668	0.380702	1	1	0	0	1465	1537	1343	1513
rs3685856	14.26738	0.022265	0	0	0	0	1465	1537	1343	1513
rs26861690	14.28965	0.199482	4	7	2	2	1465	1537	1343	1513
rs26844596	14.48913	0.193344	4	5	5	5	1465	1537	1343	1513
rs3684881	14.68247	0.220835	0	0	0	0	1465	1537	1343	1513
rs3703198	14.90331	0.19172	2	6	2	2	1465	1537	1343	1513
rs26836849	15.09503	0.241052	0	0	0	0	1465	1537	1343	1513
rs26828715	15.33608	0.128686	1	0	0	0	1465	1537	1343	1513
rs26872405	15.46477	0.399232	1	2	1	0	1465	1537	1343	1513
rs26830367	15.864	0.317193	0	0	0	0	1465	1537	1343	1513
rs3689494	16.18119	0.123139	2	1	0	1	1465	1537	1343	1513
rs26863672	16.30433	0.210241	0	0	0	0	1465	1537	1343	1513
rs26833619	16.51457	0.176635	1	0	0	0	1465	1537	1343	1513
rs26909185	16.69121	0.047735	1	0	0	1	1465	1537	1343	1513
rs26894221	16.73894	0.046124	22	23	7	8	1465	1537	1343	1513
rs26894056	16.78506	0.001826	0	0	0	0	1465	1537	1343	1513
rs26894040	16.78689	0.099877	0	0	0	1	1465	1537	1343	1513
rs26873745	16.88677	0.245591	0	0	0	0	1465	1537	1343	1513
rs26851158	17.13236	0.15154	1	0	0	0	1465	1537	1343	1513
rs26838378	17.2839	0.204906	6	4	0	1	1465	1537	1343	1513
rs26830545	17.4888	0.181625	4	2	2	0	1465	1537	1343	1513

rs26852629	17.67043	0.018739	0	0	0	0	1465	1537	1343	1513
rs26852615	17.68917	0.199655	0	0	0	0	1465	1537	1343	1513
rs26842812	17.88882	0.044806	0	0	0	0	1465	1537	1343	1513
rs26842669	17.93363	0.120597	11	13	6	5	1465	1537	1343	1513
rs26823526	18.05423	0.024858	1	1	2	1	1465	1537	1343	1513
rs26873905	18.07908	0.205818	0	0	0	0	1465	1537	1343	1513
rs26843217	18.2849	0.01998	0	0	0	0	1465	1537	1343	1513
rs26843190	18.30488	0.179807	0	1	0	0	1465	1537	1343	1513
rs26827225	18.48469	0.051206	0	0	0	0	1465	1537	1343	1513
rs26827083	18.5359	0.049252	0	0	0	0	1465	1537	1343	1513
rs26863331	18.58515	0.050138	0	0	0	0	1465	1537	1343	1513
rs26863119	18.63529	0.0268	0	0	0	0	1465	1537	1343	1513
rs26842349	18.66209	0.099012	0	0	0	0	1465	1537	1343	1513
rs26842175	18.7611	0.059885	9	12	1	2	1465	1537	1343	1513
rs26842092	18.82098	0.053034	0	0	0	0	1465	1537	1343	1513
rs26831432	18.87402	0.016771	0	0	0	0	1465	1537	1343	1513
rs26831368	18.89079	0.194521	1	4	0	0	1465	1537	1343	1513
rs26853474	19.08531	0.204205	0	1	0	0	1465	1537	1343	1513
rs26832200	19.28951	0.203157	0	0	1	0	1465	1537	1343	1513
rs26829173	19.49267	0.182183	2	1	1	0	1465	1537	1343	1513
rs26837882	19.67485	0.204824	1	1	0	0	1465	1537	1343	1513
rs3707185	19.87968	0.015928	0	0	0	0	1465	1537	1343	1513
rs26860200	19.89561	0.195	1	0	0	0	1465	1537	1343	1513
rs26830733	20.09061	0.20683	0	0	0	0	1465	1537	1343	1513
rs26837843	20.29744	0.197449	1	0	2	0	1465	1537	1343	1513
rs26823846	20.49488	0.197955	0	3	1	6	1465	1537	1343	1513
rs26864085	20.69284	0.001981	0	0	0	0	1465	1537	1343	1513
rs26864074	20.69482	0.000768	0	0	0	0	1465	1537	1343	1513
rs26864068	20.69559	0.047109	0	0	0	0	1465	1537	1343	1513
rs26864047	20.7427	0.001304	0	0	0	0	1465	1537	1343	1513
rs26864045	20.744	0.002634	0	0	0	0	1465	1537	1343	1513
rs26864039	20.74664	0.001757	0	0	0	0	1465	1537	1343	1513
rs26864031	20.74839	0.015526	0	0	0	0	1465	1537	1343	1513
rs26864012	20.76392	0.013857	2	3	16	13	1465	1537	1343	1513
rs26864008	20.77778	0.000575	0	0	1	0	1465	1537	1343	1513
rs26864006	20.77835	0.000235	0	0	0	0	1465	1537	1343	1513
rs26864003	20.77859	0.000513	0	0	0	0	1465	1537	1343	1513
rs26864000	20.7791	0.000509	0	0	1	1	1465	1537	1343	1513
rs26863996	20.77961	0.004616	0	0	1	0	1465	1537	1343	1513
rs26863993	20.78422	0.005816	0	0	0	0	1465	1537	1343	1513
rs26863975	20.79004	0.003459	1	0	1	1	1465	1537	1343	1513
rs26863969	20.7935	0.002849	0	0	0	6	1465	1537	1343	1513
rs26848056	20.79635	0.001564	0	0	0	0	1465	1537	1343	1513
rs26848045	20.79791	0.002521	0	1	0	0	1465	1537	1343	1513
rs26848031	20.80043	0.026165	2	0	9	5	1465	1537	1343	1513
rs26847992	20.8266	0.003008	0	0	0	0	1465	1537	1343	1513
rs26847982	20.82961	0.007535	0	0	0	1	1465	1537	1343	1513

rs26847971	20.83714	0.002263	1	0	1	0	1465	1537	1343	1513
rs26847960	20.8394	0.043684	0	1	1	0	1465	1537	1343	1513
rs26847914	20.88309	0.00166	0	0	0	0	1465	1537	1343	1513
rs26847910	20.88475	0.000838	0	0	0	0	1465	1537	1343	1513
rs26847906	20.88559	0.012013	0	0	0	0	1465	1537	1343	1513
rs26847902	20.8976	0.395749	1	0	1	1	1465	1537	1343	1513
rs26862245	21.29335	0.176892	0	0	0	0	1465	1537	1343	1513
rs26848213	21.47024	0.246041	0	0	0	0	1465	1537	1343	1513
rs26829988	21.71628	0.199207	0	0	0	0	1465	1537	1343	1513
rs26829844	21.91549	0.17121	0	0	2	0	1465	1537	1343	1513
rs26840258	22.0867	0.248519	0	0	0	0	1465	1537	1343	1513
rs26840006	22.33522	0.132721	2	0	0	0	1465	1537	1343	1513
rs26832688	22.46794	0.223054	0	0	2	0	1465	1537	1343	1513
rs26858845	22.69099	0.049009	0	0	2	0	1465	1537	1343	1513
rs4228622	22.74	0.118878	0	2	4	1	1465	1537	1343	1513
rs26846222	22.85888	0.046906	0	0	0	0	1465	1537	1343	1513
rs4228627	22.90578	0.037438	0	0	4	1	1465	1537	1343	1513
rs26846086	22.94322	0.029868	0	0	0	0	1465	1537	1343	1513
rs26846060	22.97309	0.073981	2	0	6	4	1465	1537	1343	1513
rs26846046	23.04707	0.115053	1	2	0	3	1465	1537	1343	1513
rs26858816	23.16212	0.102796	0	0	6	1	1465	1537	1343	1513
rs26858765	23.26492	0.025126	0	0	0	0	1465	1537	1343	1513
rs26858743	23.29005	0.200696	0	0	0	0	1465	1537	1343	1513
rs26824107	23.49074	0.183283	0	0	0	1	1465	1537	1343	1513
rs6413249	23.67403	0.26009	6	1	2	0	1465	1537	1343	1513
rs28202265	23.93412	0.155241	2	2	0	0	1465	1537	1343	1513
rs28180054	24.08936	0.201167	1	4	2	1	1465	1537	1343	1513
rs26859818	24.29052	0.095706	1	0	1	0	1465	1537	1343	1513
rs3673413	24.38623	0.153603	2	0	2	0	1465	1537	1343	1513
rs26826632	24.53983	0.15165	1	0	0	0	1465	1537	1343	1513
rs26840844	24.69148	0.199938	1	1	0	0	1465	1537	1343	1513
rs26867899	24.89142	0.049622	0	0	0	0	1465	1537	1343	1513
rs26853108	24.94104	0.04667	0	0	0	0	1465	1537	1343	1513
rs26853069	24.98771	0.034078	10	3	2	4	1465	1537	1343	1513
rs26853004	25.02179	0.063851	0	0	0	0	1465	1537	1343	1513
rs26852894	25.08564	0.00678	0	0	0	0	1465	1537	1343	1513
rs26852870	25.09242	0.196247	0	0	0	0	1465	1537	1343	1513
rs26821223	25.28867	0.197039	0	0	0	0	1465	1537	1343	1513
rs26856869	25.48571	0.207886	0	1	0	0	1465	1537	1343	1513
rs26825423	25.69359	0.065354	4	0	0	0	1465	1537	1343	1513
rs26876607	25.75895	0.061689	0	0	0	0	1465	1537	1343	1513
rs26876576	25.82064	0.043738	0	0	0	0	1465	1537	1343	1513
rs26862096	25.86437	0.055242	0	1	0	0	1465	1537	1343	1513
rs26862046	25.91962	0.045104	5	6	4	3	1465	1537	1343	1513
rs26861921	25.96472	0.052705	3	2	2	1	1465	1537	1343	1513
rs26846470	26.01743	0.072746	0	0	0	0	1465	1537	1343	1513
rs26846361	26.09017	0.202564	1	1	0	0	1465	1537	1343	1513

rs26827428	26.29274	0.205678	0	0	0	0	1465	1537	1343	1513
rs26856371	26.49841	0.190859	3	6	1	2	1465	1537	1343	1513
rs26844420	26.68927	0.055821	6	5	1	3	1465	1537	1343	1513
rs26844334	26.74509	0.060565	0	0	0	0	1465	1537	1343	1513
rs26844286	26.80566	0.063128	6	10	2	6	1465	1537	1343	1513
rs26827065	26.86879	0.027092	2	1	0	1	1465	1537	1343	1513
rs26826969	26.89588	0.190433	0	0	0	0	1465	1537	1343	1513
rs26861209	27.08631	0.214641	0	0	0	0	1465	1537	1343	1513
rs26845665	27.30095	0.1908	0	0	0	0	1465	1537	1343	1513
rs26845487	27.49175	0.207094	0	0	0	0	1465	1537	1343	1513
rs3684619	27.69885	0.172342	2	2	0	1	1465	1537	1343	1513
rs26826329	27.87119	0.219499	2	3	0	1	1465	1537	1343	1513
rs26862901	28.09069	0.397342	1	0	0	1	1465	1537	1343	1513
rs26823286	28.48803	0.195593	1	0	0	1	1465	1537	1343	1513
rs26857516	28.68362	0.25312	0	0	0	0	1465	1537	1343	1513
rs26835656	28.93674	0.154175	0	0	0	0	1465	1537	1343	1513
rs26835468	29.09092	0.21318	0	0	1	0	1465	1537	1343	1513
rs26822069	29.3041	0.216258	0	0	0	0	1465	1537	1343	1513
rs26857920	29.52036	0.404496	1	1	0	0	1465	1537	1343	1513
rs26821547	29.92485	0.127779	0	1	2	1	1465	1537	1343	1513
rs26860962	30.05263	0.006757	0	0	0	0	1465	1537	1343	1513
rs13480937	30.05939	0.087184	0	0	0	1	1465	1537	1343	1513
rs26843856	30.14657	0.046063	0	0	0	0	1465	1537	1343	1513
rs26843782	30.19263	0.049554	0	0	0	0	1465	1537	1343	1513
rs26843689	30.24219	0.025195	0	0	0	0	1465	1537	1343	1513
rs26822829	30.26738	0.038228	0	0	0	0	1465	1537	1343	1513
rs26822799	30.30561	0.111574	3	4	1	2	1465	1537	1343	1513
rs26822651	30.41719	0.001934	0	0	0	0	1465	1537	1343	1513
rs26822638	30.41912	0.03684	2	0	0	0	1465	1537	1343	1513
rs26822585	30.45596	0.035597	0	0	0	0	1465	1537	1343	1513
rs26867642	30.49156	0.001812	0	0	0	0	1465	1537	1343	1513
rs26867635	30.49337	0.002079	0	0	0	0	1465	1537	1343	1513
rs26867633	30.49545	0.226034	0	0	0	0	1465	1537	1343	1513
rs26851515	30.72148	0.16518	2	2	0	0	1465	1537	1343	1513
rs26839850	30.88666	0.170648	0	0	0	0	1465	1537	1343	1513
rs26828184	31.05731	0.204795	1	1	0	1	1465	1537	1343	1513
rs26870877	31.2621	0.181459	1	1	1	0	1465	1537	1343	1513
rs26839019	31.44356	0.207394	1	2	4	4	1465	1537	1343	1513
rs26853181	31.65096	0.234673	0	0	0	0	1465	1537	1343	1513
rs26870639	31.88563	0.185854	0	2	0	0	1465	1537	1343	1513
rs26841289	32.07148	0.058556	0	1	0	0	1465	1537	1343	1513
rs4228647	32.13004	0.162196	2	1	2	3	1465	1537	1343	1513
rs26862691	32.29224	0.201136	0	1	3	1	1465	1537	1343	1513
rs26847248	32.49337	0.199695	4	5	3	5	1465	1537	1343	1513
rs26866744	32.69307	0.198057	1	0	2	2	1465	1537	1343	1513
rs6297874	32.89112	0.060193	3	4	8	5	1465	1537	1343	1513
rs3723833	32.95132	0.143621	1	4	1	1	1465	1537	1343	1513

rs26837268	33.09494	0.20951	0	0	0	0	1465	1537	1343	1513
rs26825590	33.30445	0.202091	0	0	0	0	1465	1537	1343	1513
rs26854589	33.50654	0.052847	1	2	3	0	1465	1537	1343	1513
rs26854443	33.55939	0.060383	0	0	0	0	1465	1537	1343	1513
rs26843554	33.61977	0.03248	23	12	16	8	1465	1537	1343	1513
rs26843530	33.65225	0.0626	2	3	3	7	1465	1537	1343	1513
rs26843520	33.71485	0.160574	0	0	0	0	1465	1537	1343	1513
rs26843353	33.87542	0.214995	0	1	0	0	1465	1537	1343	1513
rs26851824	34.09042	0.19945	4	6	5	1	1465	1537	1343	1513
rs26826025	34.28987	0.146293	0	2	0	0	1465	1537	1343	1513
rs50622522	34.43616	0.237397	5	5	5	5	1465	1537	1343	1513
rs26914344	34.67356	0.17968	0	0	1	0	1465	1537	1343	1513
rs26901277	34.85324	0.20761	0	1	0	0	1465	1537	1343	1513
rs28219340	35.06085	0.050672	3	4	7	9	1465	1537	1343	1513
rs28219138	35.11152	0.050164	0	0	0	0	1465	1537	1343	1513
rs28203958	35.16168	0.038867	0	0	0	0	1465	1537	1343	1513
rs28184193	35.20055	0.041353	0	0	0	0	1465	1537	1343	1513
rs28184053	35.2419	0.012368	0	0	0	2	1465	1537	1343	1513
rs26926736	35.25427	0.05173	0	0	0	0	1465	1537	1343	1513
rs26898226	35.306	0.04883	4	3	0	1	1465	1537	1343	1513
rs26885446	35.35483	0.049615	1	5	15	6	1465	1537	1343	1513
rs26918566	35.40445	0.043881	0	0	0	0	1465	1537	1343	1513
rs26918349	35.44833	0.017062	0	0	0	0	1465	1537	1343	1513
rs26918287	35.46539	0.192814	3	1	3	1	1465	1537	1343	1513
rs26886961	35.6582	0.222638	2	2	2	3	1465	1537	1343	1513
rs26883155	35.88084	0.061746	0	0	0	0	1465	1537	1343	1513
rs3672953	35.94259	0.126638	0	3	1	1	1465	1537	1343	1513
rs26899633	36.06923	0.170838	1	3	0	0	1465	1537	1343	1513
rs26886476	36.24006	0.419748	3	1	1	1	1465	1537	1343	1513
rs26902575	36.65981	0.153369	3	4	3	5	1465	1537	1343	1513
rs26884721	36.81318	0.046886	8	14	3	7	1465	1537	1343	1513
rs26918820	36.86007	0.179919	5	3	1	5	1465	1537	1343	1513
rs26901545	37.03999	0.209652	1	2	1	1	1465	1537	1343	1513
rs26883934	37.24964	0.21499	0	1	0	0	1465	1537	1343	1513
rs26914915	37.46463	0.209674	2	1	0	0	1465	1537	1343	1513
rs26914766	37.6743	0.220519	0	0	0	0	1465	1537	1343	1513
rs29406423	37.89482	0.19126	1	0	0	0	1465	1537	1343	1513
rs3023254	38.08608	0.173962	0	2	0	0	1465	1537	1343	1513
rs28193313	38.26004	0.232115	0	0	0	0	1465	1537	1343	1513
rs28222020	38.49216	0.170034	4	3	0	0	1465	1537	1343	1513
rs28227281	38.66219	0.184541	0	0	0	0	1465	1537	1343	1513
rs28212397	38.84673	0.210129	0	1	0	0	1465	1537	1343	1513
rs28212168	39.05686	0.177346	0	0	2	1	1465	1537	1343	1513
rs28206910	39.23421	0.020109	2	1	2	3	1465	1537	1343	1513
rs28206900	39.25432	0.05839	0	0	0	0	1465	1537	1343	1513
rs28206838	39.31271	0.049434	13	18	5	8	1465	1537	1343	1513
rs28187805	39.36214	0.041533	3	7	3	11	1465	1537	1343	1513

rs28187733	39.40367	0.056819	5	0	1	1	1465	1537	1343	1513
rs28187656	39.46049	0.012971	0	0	0	0	1465	1537	1343	1513
rs28187637	39.47346	0.217612	2	0	0	1	1465	1537	1343	1513
rs28221549	39.69108	0.164065	0	0	0	0	1465	1537	1343	1513
rs28186334	39.85514	0.198546	0	0	0	0	1465	1537	1343	1513
rs28220486	40.05369	0.207663	0	0	0	0	1465	1537	1343	1513
rs28204721	40.26135	0.052827	0	0	0	1	1465	1537	1343	1513
rs28185673	40.31418	0.050152	1	2	0	0	1465	1537	1343	1513
rs28185599	40.36433	0.050564	10	7	1	3	1465	1537	1343	1513
rs28242246	40.41489	0.048256	1	2	0	0	1465	1537	1343	1513
rs28224153	40.46315	0.201167	0	0	0	0	1465	1537	1343	1513
rs28223914	40.66432	0.225986	0	0	0	0	1465	1537	1343	1513
rs28209851	40.8903	0.17063	0	1	0	0	1465	1537	1343	1513
rs28189995	41.06093	0.186197	0	0	0	0	1465	1537	1343	1513
rs28222810	41.24713	0.215547	0	1	0	0	1465	1537	1343	1513
rs28207784	41.46268	0.215861	2	2	0	0	1465	1537	1343	1513
rs28190696	41.67854	0.187363	0	0	0	0	1465	1537	1343	1513
rs28224665	41.8659	0.179848	0	0	0	0	1465	1537	1343	1513
rs28209646	42.04575	0.223382	1	1	0	1	1465	1537	1343	1513
rs28191935	42.26913	0.17158	0	0	0	0	1465	1537	1343	1513
rs3090959	42.44071	0.199132	0	0	0	0	1465	1537	1343	1513
rs28203089	42.63984	0.228584	0	0	0	0	1465	1537	1343	1513
rs28235329	42.86843	0.19953	2	1	0	0	1465	1537	1343	1513
rs28205250	43.06796	0.19308	1	1	0	0	1465	1537	1343	1513
rs28183671	43.26104	0.208833	1	4	0	0	1465	1537	1343	1513
rs28204642	43.46987	0.18899	0	0	0	0	1465	1537	1343	1513
rs28184838	43.65886	0.127218	5	3	4	2	1465	1537	1343	1513
rs3691185	43.78608	0.022043	0	0	0	0	1465	1537	1343	1513
rs28184595	43.80812	0.036057	1	0	0	0	1465	1537	1343	1513
rs26917288	43.84418	0.052854	0	0	0	0	1465	1537	1343	1513
rs26917149	43.89703	0.053712	0	0	0	0	1465	1537	1343	1513
rs26902288	43.95074	0.083999	2	11	0	0	1465	1537	1343	1513
rs26929712	44.03474	0.030618	4	4	0	0	1465	1537	1343	1513
rs26916386	44.06536	0.199548	0	0	1	0	1465	1537	1343	1513
rs3701270	44.26491	0.192473	0	1	0	0	1465	1537	1343	1513
rs6216922	44.45738	0.203918	6	3	1	1	1465	1537	1343	1513
rs26915809	44.6613	0.199034	0	1	0	3	1465	1537	1343	1513
rs26929084	44.86033	0.200791	0	1	0	0	1465	1537	1343	1513
rs26883589	45.06112	0.05354	0	1	0	0	1465	1537	1343	1513
rs26927044	45.11466	0.048108	5	9	2	7	1465	1537	1343	1513
rs26912212	45.16277	0.048894	0	0	0	0	1465	1537	1343	1513
rs26912108	45.21167	0.048967	2	1	0	0	1465	1537	1343	1513
rs26898760	45.26063	0.20168	2	3	0	1	1465	1537	1343	1513
rs26930634	45.46231	0.209383	1	0	0	0	1465	1537	1343	1513
rs6191649	45.6717	0.176681	0	0	0	0	1465	1537	1343	1513
rs26982106	45.84838	0.09775	0	1	1	1	1465	1537	1343	1513
rs26981892	45.94613	0.060206	1	0	0	0	1465	1537	1343	1513

rs26967285	46.00633	0.089369	0	0	0	0	1465	1537	1343	1513
rs28230760	46.0957	0.050177	9	6	0	2	1465	1537	1343	1513
rs26951378	46.14588	0.050267	0	1	2	1	1465	1537	1343	1513
rs26951285	46.19615	0.213285	0	1	1	3	1465	1537	1343	1513
rs28215308	46.40943	0.055371	5	3	21	19	1465	1537	1343	1513
rs28200133	46.4648	0.191538	2	3	3	5	1465	1537	1343	1513
rs26978710	46.65634	0.010524	1	0	0	0	1465	1537	1343	1513
rs3686921	46.66686	0.204338	0	0	0	0	1465	1537	1343	1513
rs26964851	46.8712	0.19857	0	0	0	0	1465	1537	1343	1513
rs26980102	47.06977	0.216928	0	1	1	0	1465	1537	1343	1513
rs26964479	47.2867	0.134	0	0	0	0	1465	1537	1343	1513
rs26951586	47.4207	0.255336	0	0	0	1	1465	1537	1343	1513
rs26973638	47.67604	0.184427	2	6	2	0	1465	1537	1343	1513
rs26948237	47.86046	0.04482	2	1	0	0	1465	1537	1343	1513
rs26948098	47.90528	0.050224	5	1	2	1	1465	1537	1343	1513
rs26990043	47.95551	0.042377	0	0	0	1	1465	1537	1343	1513
rs26974568	47.99788	0.047378	0	0	0	0	1465	1537	1343	1513
rs26974412	48.04526	0.01774	7	3	0	2	1465	1537	1343	1513
rs26960550	48.063	0.197669	0	0	0	0	1465	1537	1343	1513
rs26945797	48.26067	0.183017	0	0	0	0	1465	1537	1343	1513
rs26991809	48.44369	0.181941	2	1	3	5	1465	1537	1343	1513
rs26974844	48.62563	0.28327	0	0	1	0	1465	1537	1343	1513
rs26960150	48.9089	0.172	2	1	1	1	1465	1537	1343	1513
rs26946843	49.0809	0.164977	0	0	0	0	1465	1537	1343	1513
rs26976672	49.24588	0.038724	0	0	0	0	1465	1537	1343	1513
rs26976604	49.2846	0.041706	0	0	0	0	1465	1537	1343	1513
rs26976547	49.32631	0.047642	0	0	0	0	1465	1537	1343	1513
rs26959648	49.37395	0.048615	0	0	0	0	1465	1537	1343	1513
rs26959578	49.42256	0.037579	1	9	17	23	1465	1537	1343	1513
rs6203793	49.46014	0.19844	2	5	3	4	1465	1537	1343	1513
rs26947388	49.65858	0.048995	0	0	0	0	1465	1537	1343	1513
rs26981841	49.70758	0.043002	0	0	0	0	1465	1537	1343	1513
rs26981773	49.75058	0.055976	14	8	26	27	1465	1537	1343	1513
rs26981615	49.80656	0.059633	0	0	0	0	1465	1537	1343	1513
rs16787288	49.86619	0.194947	5	2	3	4	1465	1537	1343	1513
rs26952013	50.06114	0.202413	0	0	1	1	1465	1537	1343	1513
rs26980012	50.26355	0.202325	0	0	0	0	1465	1537	1343	1513
rs26965494	50.46587	0.092896	0	0	0	0	1465	1537	1343	1513
rs6199956	50.55877	0.098661	1	0	0	0	1465	1537	1343	1513
rs26975447	50.65743	0.2012	0	0	0	0	1465	1537	1343	1513
rs26962199	50.85863	0.190234	0	0	0	0	1465	1537	1343	1513
rs26975236	51.04886	0.041203	0	0	0	0	1465	1537	1343	1513
rs26975100	51.09007	0.048686	0	0	0	0	1465	1537	1343	1513
rs26975028	51.13875	0.05091	0	0	0	0	1465	1537	1343	1513
rs26961848	51.18966	0.060047	2	3	11	12	1465	1537	1343	1513
rs26949356	51.24971	0.049507	0	0	0	0	1465	1537	1343	1513
rs26949174	51.29922	0.041457	4	2	2	2	1465	1537	1343	1513

rs26990879	51.34067	0.058442	0	0	0	0	1465	1537	1343	1513
rs26990872	51.39912	0.041303	1	1	0	0	1465	1537	1343	1513
rs26975824	51.44042	0.030557	0	0	0	0	1465	1537	1343	1513
rs4228701	51.47098	0.190482	1	1	0	0	1465	1537	1343	1513
rs26958702	51.66146	0.204838	2	0	0	2	1465	1537	1343	1513
rs26947248	51.8663	0.196536	0	0	0	0	1465	1537	1343	1513
rs26977622	52.06283	0.214866	1	1	1	0	1465	1537	1343	1513
rs26977385	52.2777	0.182904	2	6	1	0	1465	1537	1343	1513
rs26946606	52.4606	0.20712	3	5	4	5	1465	1537	1343	1513
rs26963565	52.66772	0.099036	0	2	5	5	1465	1537	1343	1513
rs6304811	52.76676	0.050113	1	2	3	3	1465	1537	1343	1513
rs26949833	52.81687	0.044263	0	0	1	1	1465	1537	1343	1513
rs26983937	52.86113	0.192473	1	0	1	1	1465	1537	1343	1513
rs26945502	53.05361	0.168872	4	5	2	1	1465	1537	1343	1513
rs13495477	53.22248	0.236766	1	0	0	0	1465	1537	1343	1513
rs13485000	53.45925	0.200028	0	2	1	5	1465	1537	1343	1513
rs26943763	53.65927	0.183969	0	0	2	3	1465	1537	1343	1513
rs13495733	53.84324	0.219146	0	0	0	0	1465	1537	1343	1513
rs13485929	54.06239	0.072592	0	0	0	1	1465	1537	1343	1513
rs3695837	54.13498	0.328602	0	0	0	0	1465	1537	1343	1513
rs26958980	54.46358	0.198728	0	2	0	0	1465	1537	1343	1513
rs26988838	54.66231	0.201042	0	0	0	0	1465	1537	1343	1513
rs26961263	54.86335	0.198331	0	1	0	0	1465	1537	1343	1513
rs26974291	55.06168	0.198122	5	3	4	8	1465	1537	1343	1513
rs26959678	55.25981	0.193439	0	0	0	0	1465	1537	1343	1513
rs26977003	55.45324	0.410006	0	2	1	0	1465	1537	1343	1513
rs26977258	55.86325	0.205187	0	2	1	0	1465	1537	1343	1513
rs26949616	56.06844	0.05003	0	1	0	1	1465	1537	1343	1513
rs26994523	56.11847	0.049874	3	3	1	0	1465	1537	1343	1513
rs26978192	56.16834	0.05207	0	0	0	0	1465	1537	1343	1513
rs26978095	56.22041	0.048716	0	0	0	0	1465	1537	1343	1513
rs26977993	56.26913	0.0423	2	1	1	0	1465	1537	1343	1513
rs26963775	56.31143	0.018142	1	2	0	0	1465	1537	1343	1513
rs3088940	56.32957	0.12697	4	2	2	0	1465	1537	1343	1513
rs26943971	56.45654	0.402881	1	0	0	0	1465	1537	1343	1513
rs26946217	56.85942	0.211871	2	0	0	0	1465	1537	1343	1513
rs26947894	57.07129	0.053492	2	0	2	3	1465	1537	1343	1513
rs26994170	57.12478	0.00931	4	13	11	12	1465	1537	1343	1513
rs26977896	57.13409	0.104441	1	0	0	0	1465	1537	1343	1513
rs26977689	57.23853	0.027717	0	0	0	0	1465	1537	1343	1513
rs26977675	57.26625	0.071382	0	0	0	0	1465	1537	1343	1513
rs3714402	57.33763	0.106624	0	0	0	0	1465	1537	1343	1513
rs26960596	57.44426	0.282919	0	2	0	0	1465	1537	1343	1513
rs26980744	57.72718	0.119982	0	2	0	1	1465	1537	1343	1513
rs26966485	57.84716	0.299845	3	7	4	2	1465	1537	1343	1513
rs26991235	58.147	0.051094	5	4	0	2	1465	1537	1343	1513
rs26991201	58.1981	0.585419	2	3	1	0	1465	1537	1343	1513

rs3023311	58.78352	0.104502	1	2	1	2	1465	1537	1343	1513
rs26955638	58.88802	0.194073	1	0	2	3	1465	1537	1343	1513
rs26942832	59.08209	0.218669	0	2	0	1	1465	1537	1343	1513
rs28226166	59.30076	0.1857	1	4	1	2	1465	1537	1343	1513
rs28211297	59.48646	0.184958	0	0	0	0	1465	1537	1343	1513
rs26971347	59.67142	0.224929	2	1	1	2	1465	1537	1343	1513
rs26941519	59.89635	0.049837	3	1	8	18	1465	1537	1343	1513
rs26941421	59.94618	0.050379	0	1	1	0	1465	1537	1343	1513
rs26987598	59.99656	0.04958	0	1	0	0	1465	1537	1343	1513
rs26973051	60.04614	0.042024	0	0	0	0	1465	1537	1343	1513
rs26972887	60.08817	0.01248	0	0	0	0	1465	1537	1343	1513
rs26972883	60.10065	0.182475	0	0	0	0	1465	1537	1343	1513
rs26986414	60.28312	0.222602	0	1	3	5	1465	1537	1343	1513
rs26972053	60.50572	0.171579	0	2	1	1	1465	1537	1343	1513
rs6410683	60.6773	0.206849	0	1	1	0	1465	1537	1343	1513
rs3023266	60.88415	0.00352	0	1	0	1	1465	1537	1343	1513
rs28233913	60.88767	0.401198	2	1	5	7	1465	1537	1343	1513
rs26941259	61.28887	0.051477	0	0	0	0	1465	1537	1343	1513
rs26941121	61.34035	0.045641	0	0	0	0	1465	1537	1343	1513
rs26985791	61.38599	0.054318	0	0	0	0	1465	1537	1343	1513
rs26969829	61.44031	0.043933	0	0	0	0	1465	1537	1343	1513
rs26969781	61.48424	0.016932	1	1	1	11	1465	1537	1343	1513
rs26969758	61.50117	0.19079	1	1	3	10	1465	1537	1343	1513
rs26955011	61.69196	0.392638	2	1	4	0	1465	1537	1343	1513
rs26954166	62.0846	0.207888	0	0	0	0	1465	1537	1343	1513
rs26924979	62.29249	0.218614	3	3	4	5	1465	1537	1343	1513
rs6288504	62.5111	0.176009	0	1	5	4	1465	1537	1343	1513
rs26941028	62.68711	0.20186	0	0	0	1	1465	1537	1343	1513
rs26958056	62.88897	0.003089	0	0	1	1	1465	1537	1343	1513
rs26958045	62.89206	0.003213	2	1	16	19	1465	1537	1343	1513
rs26958027	62.89527	0.012722	0	1	1	3	1465	1537	1343	1513
rs26958015	62.90799	0.001119	0	0	0	0	1465	1537	1343	1513
rs26958010	62.90911	0.00135	0	0	0	0	1465	1537	1343	1513
rs3024185	62.91046	0.213919	0	0	0	0	1465	1537	1343	1513
rs26940006	63.12438	0.156147	1	1	0	0	1465	1537	1343	1513
rs26956868	63.28053	0.147778	0	1	1	1	1465	1537	1343	1513
rs3705233	63.42831	0.26071	7	7	3	6	1465	1537	1343	1513
rs26969493	63.68902	0.200917	3	1	0	1	1465	1537	1343	1513
rs26939373	63.88993	0.405712	3	0	3	3	1465	1537	1343	1513
rs26958257	64.29565	0	0	0	0	0	1465	1537	1343	1513
rs26940697	64.29565	0.190712	0	2	0	0	1465	1537	1343	1513
rs26967997	64.48636	0.189936	4	3	2	2	1465	1537	1343	1513
rs3023989	64.67629	0.209254	4	4	1	1	1465	1537	1343	1513
rs26905668	64.88555	0.097759	4	0	1	1	1465	1537	1343	1513
rs26971032	64.98331	0.050612	0	0	0	0	1465	1537	1343	1513
rs26970774	65.03392	0.045272	0	0	0	0	1465	1537	1343	1513
rs26955810	65.07919	0.0337	0	0	0	0	1465	1537	1343	1513

rs26940413	65.11289	0.047336	2	3	0	1	1465	1537	1343	1513
rs26940289	65.16023	0.048701	0	0	0	0	1465	1537	1343	1513
rs26940200	65.20893	0.050575	1	1	0	0	1465	1537	1343	1513
rs26968658	65.2595	0.01539	4	0	0	0	1465	1537	1343	1513
rs26968616	65.27489	0.248985	0	0	0	0	1465	1537	1343	1513
rs3023267	65.52388	0.156144	0	0	0	0	1465	1537	1343	1513
rs26921482	65.68002	0.214215	1	1	0	0	1465	1537	1343	1513
rs26907214	65.89424	0.194335	0	0	0	0	1465	1537	1343	1513
rs26938421	66.08857	0.196945	1	4	0	0	1465	1537	1343	1513
rs26908519	66.28552	0.049648	3	4	4	3	1465	1537	1343	1513
rs26891552	66.33517	0.142763	0	3	5	4	1465	1537	1343	1513
rs26923314	66.47793	0.115399	0	2	0	0	1465	1537	1343	1513
rs3655482	66.59333	0.092978	1	0	0	0	1465	1537	1343	1513
rs26906927	66.68631	0.203058	1	3	2	0	1465	1537	1343	1513
rs26922427	66.88936	0.201838	2	0	1	1	1465	1537	1343	1513
rs26892244	67.0912	0.135543	8	6	4	2	1465	1537	1343	1513
rs26907958	67.22674	0.061592	4	6	2	3	1465	1537	1343	1513
rs26907810	67.28834	0.202691	1	1	1	0	1465	1537	1343	1513
rs28233620	67.49103	0.198262	0	0	0	0	1465	1537	1343	1513
rs28201481	67.68929	0.198372	8	6	3	2	1465	1537	1343	1513
rs26919501	67.88766	0.198062	0	0	0	0	1465	1537	1343	1513
rs26892508	68.08572	0.20327	0	0	0	0	1465	1537	1343	1513
rs26906546	68.28899	0.201775	1	1	1	2	1465	1537	1343	1513
rs26893510	68.49077	0.200096	1	1	0	0	1465	1537	1343	1513
rs26921965	68.69086	0.167891	0	1	0	0	1465	1537	1343	1513
rs6178860	68.85876	0.452585	0	1	2	1	1465	1537	1343	1513
rs26922699	69.31134	0.249625	1	0	2	2	1465	1537	1343	1513
rs3668244	69.56097	0.125037	0	0	0	0	1465	1537	1343	1513
rs26908149	69.686	0.400373	1	3	1	4	1465	1537	1343	1513
rs26905062	70.08638	0.215566	0	0	0	0	1465	1537	1343	1513
rs26890843	70.30194	0.157774	0	0	1	2	1465	1537	1343	1513
rs26924501	70.45972	0.232429	3	3	2	1	1465	1537	1343	1513
rs26908879	70.69214	0.218467	0	0	0	0	1465	1537	1343	1513
rs3142844	70.91061	0.181454	0	0	0	0	1465	1537	1343	1513
rs28233165	71.09207	0.213903	0	1	0	1	1465	1537	1343	1513
rs28217621	71.30597	0.381183	2	0	2	0	1465	1537	1343	1513
rs26890617	71.68715	0.2013	2	2	1	1	1465	1537	1343	1513
rs26902768	71.88845	0.093141	0	0	0	0	1465	1537	1343	1513
rs3023278	71.98159	0.102895	0	0	0	0	1465	1537	1343	1513
rs26915220	72.08449	0.208353	1	3	0	2	1465	1537	1343	1513
rs26900719	72.29284	0.171371	1	5	0	0	1465	1537	1343	1513
rs26887964	72.46421	0.302772	0	0	0	1	1465	1537	1343	1513
rs26917916	72.76698	0.142475	4	1	2	1	1465	1537	1343	1513
rs26903480	72.90946	0.151917	1	3	5	1	1465	1537	1343	1513
rs26888927	73.06138	1.235139	1	2	0	0	1465	1537	1343	1513
rs26934776	74.29651	0.188733	0	0	0	0	1465	1537	1343	1513
rs26904676	74.48525	0.004241	0	0	0	0	1465	1537	1343	1513

rs3691128	74.48949	0.19836	0	2	0	0	1465	1537	1343	1513
rs26888818	74.68785	0.199519	0	0	0	1	1465	1537	1343	1513
rs28232537	74.88737	0.205757	0	0	0	0	1465	1537	1343	1513
rs6219537	75.09312	0.181766	0	0	0	1	1465	1537	1343	1513
rs6396927	75.27489	0.144074	0	0	0	0	1465	1537	1343	1513
rs3714172	75.41896	0.271905	0	0	0	0	1465	1537	1343	1513
rs26889532	75.69087	0.20494	0	0	0	2	1465	1537	1343	1513
rs28216357	75.89581	0.198759	1	0	0	0	1465	1537	1343	1513
rs28199705	76.09457	0.194229	3	4	1	1	1465	1537	1343	1513
rs28215706	76.2888	0.092143	0	0	0	0	1465	1537	1343	1513
rs3658198	76.38094	0.153657	0	1	1	0	1465	1537	1343	1513
rs6280242	76.5346	0.148832	0	2	0	0	1465	1537	1343	1513
rs28245795	76.68343	0.201763	0	0	0	0	1465	1537	1343	1513
rs28226689	76.88519	0.206369	0	4	0	0	1465	1537	1343	1513
rs28211842	77.09156	0.19498	0	0	0	0	1465	1537	1343	1513
rs28211669	77.28654	0.191278	4	0	0	0	1465	1537	1343	1513
rs28194740	77.47782	0.201991	0	0	0	1	1465	1537	1343	1513
rs28194615	77.67981	0.208077	0	0	0	0	1465	1537	1343	1513
rs28194495	77.88789	0.202982	2	2	6	4	1465	1537	1343	1513
rs28230041	78.09087	0.194101	0	1	0	0	1465	1537	1343	1513
rs28214837	78.28497	0.211951	0	1	0	0	1465	1537	1343	1513
rs28198949	78.49692	0.200568	1	0	0	0	1465	1537	1343	1513
rs6297515	78.69749	0.051804	2	1	1	1	1465	1537	1343	1513
rs28228061	78.74929	0.049617	2	1	5	5	1465	1537	1343	1513
rs28227907	78.79891	0.039345	0	2	0	0	1465	1537	1343	1513
rs28227849	78.83826	0.049849	0	1	1	1	1465	1537	1343	1513
rs28227790	78.8881	0.187259	4	3	2	2	1465	1537	1343	1513
rs28196795	79.07536	0.214693	0	0	0	0	1465	1537	1343	1513
rs28228940	79.29006	0.197719	8	4	3	5	1465	1537	1343	1513
rs28214187	79.48778	0.165567	0	2	1	0	1465	1537	1343	1513
rs3700839	79.65334	0.030597	0	0	0	0	1465	1537	1343	1513
rs28197279	79.68394	0.198353	0	0	0	0	1465	1537	1343	1513
rs28227682	79.88229	0.197443	0	2	1	0	1465	1537	1343	1513
rs28213038	80.07974	0.228696	4	5	1	2	1465	1537	1343	1513
rs28197119	80.30843	0.171412	0	1	0	1	1465	1537	1343	1513
rs28228240	80.47984	0.00433	0	0	0	0	1465	1537	1343	1513
rs3688710	80.48417	0.203601	3	1	0	1	1465	1537	1343	1513
rs28213436	80.68777	0.200037	0	0	0	0	1465	1537	1343	1513
rs28227048	80.88781	0.205896	1	1	0	1	1465	1537	1343	1513
rs28195710	81.09371	0.090706	0	2	0	0	1465	1537	1343	1513
rs28219735	81.18441	0.048436	0	0	0	0	1465	1537	1343	1513
rs28205525	81.23285	0.054853	0	0	0	0	1465	1537	1343	1513
rs28205432	81.2877	0.048668	0	0	0	0	1465	1537	1343	1513
rs28205268	81.33637	0.047955	0	0	0	0	1465	1537	1343	1513
rs28186593	81.38433	0.048292	2	0	0	0	1465	1537	1343	1513
rs28186386	81.43262	0.05022	10	5	0	0	1465	1537	1343	1513
rs28223834	81.48284	0.060611	0	0	0	0	1465	1537	1343	1513

rs28223673	81.54345	0.029085	0	0	0	0	1465	1537	1343	1513
rs28208836	81.57253	0.049264	0	1	0	0	1465	1537	1343	1513
rs28208753	81.6218	0.050197	0	0	0	0	1465	1537	1343	1513
rs28208584	81.67199	0.016246	0	3	0	1	1465	1537	1343	1513
rs28192297	81.68824	0.197396	4	4	1	0	1465	1537	1343	1513
rs28207602	81.88564	0.192284	2	4	2	1	1465	1537	1343	1513
rs28188663	82.07792	0.218515	0	0	0	0	1465	1537	1343	1513
rs28221212	82.29644	0.186207	0	0	0	0	1465	1537	1343	1513
rs28187045	82.48264	0.192981	2	2	0	0	1465	1537	1343	1513
rs28225369	82.67562	0.415692	0	0	0	0	1465	1537	1343	1513
rs28226327	83.09132	0.197643	0	1	0	1	1465	1537	1343	1513
rs28190482	83.28896	0.042498	0	2	0	0	1465	1537	1343	1513
rs28190438	83.33146	0.050392	0	0	0	0	1465	1537	1343	1513
rs3141846	83.38185	0.049715	1	1	6	11	1465	1537	1343	1513
rs28223562	83.43156	0.049187	0	0	1	2	1465	1537	1343	1513
rs3671906	83.48075	0.00109	0	0	0	0	1465	1537	1343	1513
rs28223402	83.48184	0.201702	0	0	0	0	1465	1537	1343	1513
rs28191710	83.68354	0.206146	0	0	0	0	1465	1537	1343	1513
rs28225717	83.88969	0.106744	1	1	0	2	1465	1537	1343	1513
rs3089858	83.99643	0.291905	0	0	0	0	1465	1537	1343	1513
rs28224204	84.28834	0.411398	1	0	0	0	1465	1537	1343	1513
rs26953550	84.69974	0.388285	3	0	1	0	1465	1537	1343	1513
rs26965244	85.08802	0.20194	0	0	1	0	1465	1537	1343	1513
rs26995728	85.28996	0.178989	0	0	0	0	1465	1537	1343	1513
rs26965902	85.46895	0.207807	0	0	0	0	1465	1537	1343	1513
rs26952912	85.67676	0.189025	1	5	4	1	1465	1537	1343	1513
rs27014738	85.86578	0.220864	0	0	1	0	1465	1537	1343	1513
rs24884009	86.08665	0.223425	1	0	1	0	1465	1537	1343	1513
rs27027078	86.31007	0.18278	0	0	0	0	1465	1537	1343	1513
rs27012057	86.49285	0.195029	0	2	2	0	1465	1537	1343	1513
rs26986792	86.68788	0.200027	1	1	0	0	1465	1537	1343	1513
rs27032064	86.88791	0.193441	0	0	0	0	1465	1537	1343	1513
rs27014056	87.08135	0.224639	0	0	0	0	1465	1537	1343	1513
rs26998456	87.30599	0.174064	3	0	0	0	1465	1537	1343	1513
rs28278405	87.48005	0.134375	1	2	0	0	1465	1537	1343	1513
rs3090226	87.61443	0.062676	4	1	2	1	1465	1537	1343	1513
rs28262760	87.6771	0.02668	2	2	5	2	1465	1537	1343	1513
rs28262667	87.70378	0.066295	7	3	4	4	1465	1537	1343	1513
rs28262626	87.77008	0.123498	1	0	2	0	1465	1537	1343	1513
rs28241129	87.89357	0.11246	2	4	0	0	1465	1537	1343	1513
rs26993948	88.00603	0.100954	0	0	0	0	1465	1537	1343	1513
rs26979507	88.10699	0.098858	0	1	0	0	1465	1537	1343	1513
rs26953339	88.20585	0.100003	0	0	0	0	1465	1537	1343	1513
rs26982404	88.30585	0.029975	0	0	0	0	1465	1537	1343	1513
rs3688569	88.33582	0.200928	7	0	1	1	1465	1537	1343	1513
rs27065246	88.53675	0.010252	9	0	0	0	1465	1537	1343	1513
rs27065208	88.547	0.317535	3	3	0	1	1465	1537	1343	1513

rs27099888	88.86454	0.101246	0	1	0	0	1465	1537	1343	1513
rs27080116	88.96579	0.056871	18	14	3	6	1465	1537	1343	1513
rs27080008	89.02266	0.050519	9	14	4	3	1465	1537	1343	1513
rs27064231	89.07318	0.041172	0	0	0	0	1465	1537	1343	1513
rs27064192	89.11435	0.130736	1	4	0	1	1465	1537	1343	1513
rs27111797	89.24508	0.058744	1	3	0	0	1465	1537	1343	1513
rs27094854	89.30383	0.010446	0	1	0	0	1465	1537	1343	1513
rs3682081	89.31427	0.020838	0	0	0	0	1465	1537	1343	1513
rs27094749	89.33511	0.058968	1	2	0	2	1465	1537	1343	1513
rs27079057	89.39408	0.041372	0	0	0	0	1465	1537	1343	1513
rs27078979	89.43545	0.0229	0	0	0	0	1465	1537	1343	1513
rs27078909	89.45835	0.004634	0	1	0	0	1465	1537	1343	1513
rs27078903	89.46299	0.003322	0	0	0	0	1465	1537	1343	1513
rs27078890	89.46631	0.001766	0	0	0	0	1465	1537	1343	1513
rs27078881	89.46807	0.002751	0	0	0	0	1465	1537	1343	1513
rs27078874	89.47082	0.002817	11	21	8	27	1465	1537	1343	1513
rs27078872	89.47364	0.001701	3	3	2	0	1465	1537	1343	1513
rs27078869	89.47534	0.012497	1	0	0	0	1465	1537	1343	1513
rs27078854	89.48784	0.050551	8	2	1	2	1465	1537	1343	1513
rs27063629	89.53839	0.203673	5	2	2	0	1465	1537	1343	1513
rs27083699	89.74206	0.203115	1	1	0	0	1465	1537	1343	1513
rs27091912	89.94518	0.161394	6	6	1	1	1465	1537	1343	1513
rs27060006	90.10657	0.233571	4	3	1	0	1465	1537	1343	1513
rs27075923	90.34014	0.393055	1	1	0	0	1465	1537	1343	1513
rs27061229	90.7332	0.163972	0	0	0	0	1465	1537	1343	1513
rs27095113	90.89717	0.099337	4	3	1	0	1465	1537	1343	1513
rs27078718	90.99651	0.100051	0	0	0	0	1465	1537	1343	1513
rs27066096	91.09656	0.09947	0	0	0	0	1465	1537	1343	1513
rs27099132	91.19603	0.100144	2	0	0	0	1465	1537	1343	1513
rs27098975	91.29617	0.069477	0	0	0	0	1465	1537	1343	1513
rs27098918	91.36565	0.174314	6	3	0	2	1465	1537	1343	1513
rs27067736	91.53996	0.228936	0	2	1	0	1465	1537	1343	1513
rs27080410	91.7689	0.146316	1	0	0	0	1465	1537	1343	1513
rs27062798	91.91522	0.226721	0	0	0	0	1465	1537	1343	1513
rs27062599	92.14194	0.186254	0	1	0	0	1465	1537	1343	1513
rs27092052	92.32819	0.226536	0	1	0	0	1465	1537	1343	1513
rs27115982	92.55473	0.187819	1	1	0	0	1465	1537	1343	1513
rs27082183	92.74255	0.049076	1	0	0	0	1465	1537	1343	1513
rs27067012	92.79162	0.054187	1	0	0	0	1465	1537	1343	1513
rs27066824	92.84581	0.048276	4	4	0	0	1465	1537	1343	1513
rs27092464	92.89408	0.036509	3	4	0	0	1465	1537	1343	1513
rs27092326	92.93059	0.188966	0	1	0	0	1465	1537	1343	1513
rs27060889	93.11956	0.21484	3	1	0	0	1465	1537	1343	1513
rs27104938	93.3344	0.050648	9	11	1	1	1465	1537	1343	1513
rs27090109	93.38505	0.050021	0	0	0	0	1465	1537	1343	1513
rs27089942	93.43507	0.105307	3	3	0	0	1465	1537	1343	1513
rs27060605	93.54038	0.050562	1	0	0	0	1465	1537	1343	1513

rs28291072	93.59094	0.049774	0	0	0	0	1465	1537	1343	1513
rs28275943	93.64071	0.055402	0	0	0	0	1465	1537	1343	1513
rs28275783	93.69611	0.070118	0	1	0	0	1465	1537	1343	1513
rs28260396	93.76623	0.165818	0	0	0	0	1465	1537	1343	1513
rs28237863	93.93205	0.198187	0	1	0	0	1465	1537	1343	1513
rs27091462	94.13024	0.209787	7	7	7	3	1465	1537	1343	1513
rs27059794	94.34002	0.193821	2	0	0	0	1465	1537	1343	1513
rs27090399	94.53384	0.126699	3	2	0	2	1465	1537	1343	1513
rs27075378	94.66054	0.07424	8	5	8	4	1465	1537	1343	1513
rs27075168	94.73478	0.200924	5	3	0	1	1465	1537	1343	1513
rs27086788	94.93571	0.05933	6	3	2	0	1465	1537	1343	1513
rs27086637	94.99504	0.049679	1	0	1	0	1465	1537	1343	1513
rs27070924	95.04472	0.050385	0	0	0	0	1465	1537	1343	1513
rs27070785	95.0951	0.037198	3	10	3	8	1465	1537	1343	1513
rs27070685	95.1323	0.20789	0	1	0	0	1465	1537	1343	1513
rs27087638	95.34019	0.194816	0	0	0	1	1465	1537	1343	1513
rs27055604	95.53501	0.052645	0	0	0	0	1465	1537	1343	1513
rs27055467	95.58765	0.044614	0	0	0	0	1465	1537	1343	1513
rs27101935	95.63226	0.036548	10	4	0	4	1465	1537	1343	1513
rs27087163	95.66881	0.050293	0	0	0	0	1465	1537	1343	1513
rs27087076	95.71911	0.00887	0	0	0	0	1465	1537	1343	1513
rs27087042	95.72798	0.207684	4	7	1	0	1465	1537	1343	1513
rs27071378	95.93566	0.051818	3	1	0	0	1465	1537	1343	1513
rs27071344	95.98748	0.050549	0	4	2	6	1465	1537	1343	1513
rs27045701	96.03803	0.074246	1	0	0	0	1465	1537	1343	1513
rs27045569	96.11227	0.051699	0	0	0	0	1465	1537	1343	1513
rs27045538	96.16397	0.055285	3	4	4	1	1465	1537	1343	1513
rs27045506	96.21926	0.045875	0	0	0	0	1465	1537	1343	1513
rs27089261	96.26513	0.049501	0	0	0	0	1465	1537	1343	1513
rs27089128	96.31463	0.022946	1	0	0	0	1465	1537	1343	1513
rs27089114	96.33758	0.197724	6	3	3	0	1465	1537	1343	1513
rs27052923	96.5353	0.398784	2	2	2	0	1465	1537	1343	1513
rs27041550	96.93409	0.193913	0	0	0	0	1465	1537	1343	1513
rs27026520	97.128	0.20922	0	0	0	0	1465	1537	1343	1513
rs27011419	97.33722	0.076039	0	0	0	0	1465	1537	1343	1513
rs13481176	97.41326	0.124072	4	4	2	1	1465	1537	1343	1513
rs27089818	97.53733	0.202041	0	1	2	1	1465	1537	1343	1513
rs27074137	97.73937	0.197609	0	0	0	0	1465	1537	1343	1513
rs27056927	97.93698	0.405862	0	0	0	0	1465	1537	1343	1513
rs13481179	98.34284	0.194282	0	0	0	0	1465	1537	1343	1513
rs27041265	98.53712	0.201469	2	0	6	5	1465	1537	1343	1513
rs27026195	98.73859	0.196436	1	0	0	0	1465	1537	1343	1513
rs27087343	98.93503	0.044435	0	3	1	0	1465	1537	1343	1513
rs27087267	98.97946	0.05045	10	9	19	19	1465	1537	1343	1513
rs27071852	99.02991	0.049091	0	0	0	0	1465	1537	1343	1513
rs27071698	99.07901	0.036274	0	0	0	0	1465	1537	1343	1513
rs27053258	99.11528	0.029222	0	0	0	0	1465	1537	1343	1513

rs27053231	99.1445	0.191495	0	0	0	1	1465	1537	1343	1513
rs27073312	99.336	0.105455	1	1	0	0	1465	1537	1343	1513
rs4229088	99.44145	0.094301	1	0	0	0	1465	1537	1343	1513
rs27074725	99.53575	0.202224	5	6	3	1	1465	1537	1343	1513
rs27103456	99.73798	0.186138	0	0	0	0	1465	1537	1343	1513
rs27072582	99.92411	0.042934	5	8	0	0	1465	1537	1343	1513
rs27057693	99.96705	0.044089	2	0	0	0	1465	1537	1343	1513
rs27057515	100.0111	0.05012	0	0	0	0	1465	1537	1343	1513
rs27088324	100.0613	0.067399	0	0	0	1	1465	1537	1343	1513
rs26979100	100.1287	0.207707	0	0	0	0	1465	1537	1343	1513
rs27058898	100.3364	0.198533	2	1	2	2	1465	1537	1343	1513
rs27075662	100.5349	0.020941	0	0	0	0	1465	1537	1343	1513
rs27075630	100.5558	0.067812	1	1	2	4	1465	1537	1343	1513
rs27075543	100.6236	0.049658	2	3	6	14	1465	1537	1343	1513
rs27058261	100.6733	0.049776	0	0	0	0	1465	1537	1343	1513
rs27058124	100.7231	0.019295	0	0	0	1	1465	1537	1343	1513
rs27058077	100.7424	0.050564	0	0	0	0	1465	1537	1343	1513
rs27089540	100.7929	0.141588	2	1	0	1	1465	1537	1343	1513
rs27089335	100.9345	0.045248	5	4	3	4	1465	1537	1343	1513
rs27073645	100.9798	0.056488	2	1	1	2	1465	1537	1343	1513
rs27073546	101.0363	0.046722	3	0	0	0	1465	1537	1343	1513
rs27073488	101.083	0.052488	0	0	0	0	1465	1537	1343	1513
rs27073459	101.1355	0.210259	0	2	2	3	1465	1537	1343	1513
rs28288744	101.3457	0.041191	0	0	0	0	1465	1537	1343	1513
rs28288691	101.3869	0.050427	0	0	0	0	1465	1537	1343	1513
rs28273224	101.4374	0.00457	0	0	0	0	1465	1537	1343	1513
rs13473438	101.4419	0.002234	0	0	0	0	1465	1537	1343	1513
rs28273188	101.4442	0.001773	0	0	0	0	1465	1537	1343	1513
rs28273179	101.4459	0.001968	0	0	0	0	1465	1537	1343	1513
rs28273168	101.4479	0.00321	0	0	0	0	1465	1537	1343	1513
rs28273161	101.4511	0.003942	0	1	0	0	1465	1537	1343	1513
rs28273145	101.4551	0.024565	2	3	6	5	1465	1537	1343	1513
rs28257432	101.4796	0.007002	0	0	0	0	1465	1537	1343	1513
rs28257425	101.4866	0.049144	0	0	1	1	1465	1537	1343	1513
rs28257250	101.5358	0.057223	0	0	0	0	1465	1537	1343	1513
rs28234943	101.593	0.043978	2	5	0	0	1465	1537	1343	1513
rs28234774	101.637	0.041464	0	0	0	0	1465	1537	1343	1513
rs28234718	101.6784	0.051017	0	0	0	0	1465	1537	1343	1513
rs27049718	101.7294	0.006219	0	1	0	0	1465	1537	1343	1513
rs27049690	101.7357	0.402027	2	1	2	2	1465	1537	1343	1513
rs27040573	102.1377	0.199433	0	0	0	0	1465	1537	1343	1513
rs27025344	102.3371	0.198444	0	0	0	0	1465	1537	1343	1513
rs27025098	102.5356	0.209472	7	3	0	3	1465	1537	1343	1513
rs27053934	102.745	0.100805	2	0	0	0	1465	1537	1343	1513
rs27019897	102.8458	0.093971	4	4	2	0	1465	1537	1343	1513
rs27007918	102.9398	0.104696	4	6	1	5	1465	1537	1343	1513
rs27007781	103.0445	0.090262	0	0	0	0	1465	1537	1343	1513

rs27036243	103.1348	0.115836	0	0	0	0	1465	1537	1343	1513
rs3687284	103.2506	0.289247	1	1	1	0	1465	1537	1343	1513
rs27021755	103.5399	0.396062	4	4	2	5	1465	1537	1343	1513
rs6335689	103.9359	0.257599	1	2	0	5	1465	1537	1343	1513
rs3153215	104.1935	0.190763	0	1	0	0	1465	1537	1343	1513
rs3024066	104.3843	0.172261	2	1	2	1	1465	1537	1343	1513
rs27038344	104.5565	0.183949	0	0	0	0	1465	1537	1343	1513
rs27023409	104.7405	0.202056	0	2	0	0	1465	1537	1343	1513
rs27005445	104.9425	0.186084	0	0	3	3	1465	1537	1343	1513
rs27055727	105.1286	0.207085	1	1	0	0	1465	1537	1343	1513
rs6236277	105.3357	0.053864	0	1	7	1	1465	1537	1343	1513
rs27025670	105.3896	0.045813	6	7	13	5	1465	1537	1343	1513
rs27009976	105.4354	0.049722	0	1	0	0	1465	1537	1343	1513
rs27009941	105.4851	0.044466	0	0	0	1	1465	1537	1343	1513
rs6315099	105.5296	0.207766	0	0	0	0	1465	1537	1343	1513
rs27038805	105.7373	0.199414	0	0	0	0	1465	1537	1343	1513
rs6274935	105.9368	0.201952	0	0	0	0	1465	1537	1343	1513
rs27010729	106.1387	0.048376	3	1	0	2	1465	1537	1343	1513
rs27048191	106.1871	0.050549	5	3	3	10	1465	1537	1343	1513
rs27033018	106.2376	0.000796	0	0	0	0	1465	1537	1343	1513
rs26979052	106.2384	0.037494	0	0	0	1	1465	1537	1343	1513
rs27032912	106.2759	0.01082	0	0	0	0	1465	1537	1343	1513
rs27018737	106.2867	0.044134	0	0	0	0	1465	1537	1343	1513
rs27018603	106.3309	0.049328	2	0	0	0	1465	1537	1343	1513
rs27018519	106.3802	0.049121	0	0	1	0	1465	1537	1343	1513
rs27004495	106.4293	0.065332	1	1	6	13	1465	1537	1343	1513
rs27004455	106.4947	0.034353	0	6	2	4	1465	1537	1343	1513
rs27056312	106.529	0.208485	0	0	0	1	1465	1537	1343	1513
rs27024736	106.7375	0.016029	0	0	0	0	1465	1537	1343	1513
rs3705163	106.7535	0.18403	0	0	0	0	1465	1537	1343	1513
rs27024502	106.9376	0.212666	1	1	0	0	1465	1537	1343	1513
rs27050302	107.1502	0.191184	2	5	4	7	1465	1537	1343	1513
rs27020459	107.3414	0.19482	0	1	1	0	1465	1537	1343	1513
rs27055093	107.5362	0.199751	3	3	0	1	1465	1537	1343	1513
rs27011066	107.736	0.051248	5	3	12	17	1465	1537	1343	1513
rs27034656	107.7872	0.050902	18	6	22	25	1465	1537	1343	1513
rs27034400	107.8381	0.050862	2	0	1	4	1465	1537	1343	1513
rs27017984	107.889	0.018342	0	0	0	0	1465	1537	1343	1513
rs27017889	107.9073	0.047724	0	0	0	0	1465	1537	1343	1513
rs27005804	107.9551	0.180993	0	0	0	0	1465	1537	1343	1513
rs27022026	108.1361	0.592409	0	0	3	2	1465	1537	1343	1513
rs27006688	108.7285	0.207244	3	4	0	2	1465	1537	1343	1513
rs27053286	108.9357	0.201325	0	0	0	0	1465	1537	1343	1513
rs27022854	109.137	0.201166	4	5	4	5	1465	1537	1343	1513
rs27004763	109.3382	0.198165	2	1	1	2	1465	1537	1343	1513
rs27035943	109.5364	0.00653	0	0	0	0	1465	1537	1343	1513
rs27035928	109.5429	0.01367	0	0	0	1	1465	1537	1343	1513

rs27035921	109.5566	0.02933	1	0	1	2	1465	1537	1343	1513
rs27022366	109.5859	0.051076	2	2	5	0	1465	1537	1343	1513
rs27022195	109.637	0.002698	0	0	0	0	1465	1537	1343	1513
rs27022190	109.6397	0.002439	0	0	0	0	1465	1537	1343	1513
rs27022181	109.6421	0.003457	0	0	0	0	1465	1537	1343	1513
rs27022176	109.6456	0.006291	0	0	2	1	1465	1537	1343	1513
rs27022152	109.6519	0.04363	0	0	0	0	1465	1537	1343	1513
rs27007359	109.6955	0.041321	0	0	1	0	1465	1537	1343	1513
rs27007273	109.7368	0.209982	1	0	5	1	1465	1537	1343	1513
rs27050015	109.9468	0.190591	0	3	0	0	1465	1537	1343	1513
rs27019267	110.1374	0.182043	3	1	1	1	1465	1537	1343	1513
rs27003729	110.3194	0.195826	0	1	0	0	1465	1537	1343	1513
rs27033429	110.5152	0.220966	4	1	6	3	1465	1537	1343	1513
rs27019523	110.7362	0.218151	1	2	0	0	1465	1537	1343	1513
rs27030121	110.9544	0.184376	2	0	0	0	1465	1537	1343	1513
rs27016458	111.1387	0.19696	1	1	2	1	1465	1537	1343	1513
rs27055978	111.3357	0.202473	0	0	2	1	1465	1537	1343	1513
rs27023631	111.5382	0.198219	0	0	0	0	1465	1537	1343	1513
rs27037562	111.7364	0.192538	0	0	0	0	1465	1537	1343	1513
rs27020090	111.9289	0.047869	10	14	8	3	1465	1537	1343	1513
rs27007648	111.9768	0.049702	0	0	0	0	1465	1537	1343	1513
rs27007453	112.0265	0.053688	0	0	0	0	1465	1537	1343	1513
rs27046693	112.0802	0.052612	0	0	0	0	1465	1537	1343	1513
rs27031262	112.1328	0.044037	0	0	0	0	1465	1537	1343	1513
rs27031117	112.1768	0.016321	0	0	0	0	1465	1537	1343	1513
rs27031076	112.1932	0.008949	0	0	0	0	1465	1537	1343	1513
rs27017223	112.2021	0.006133	0	3	1	1	1465	1537	1343	1513
rs27017204	112.2082	0.008747	0	0	0	0	1465	1537	1343	1513
rs3687980	112.217	0.01952	3	11	5	4	1465	1537	1343	1513
rs27017081	112.2365	0.024774	0	0	1	1	1465	1537	1343	1513
rs27017042	112.2613	0.028275	5	11	3	3	1465	1537	1343	1513
rs27002238	112.2896	0.01893	1	0	0	0	1465	1537	1343	1513
rs26979036	112.3085	0.042432	25	17	1	7	1465	1537	1343	1513
rs27002118	112.3509	0.177109	6	1	0	0	1465	1537	1343	1513
rs27003337	112.528	0.216024	2	0	0	1	1465	1537	1343	1513
rs27016167	112.744	0.049072	5	2	1	0	1465	1537	1343	1513
rs27001816	112.7931	0.049111	0	0	0	0	1465	1537	1343	1513
rs27048536	112.8422	0.050672	0	0	4	2	1465	1537	1343	1513
rs27033709	112.8929	0.059241	5	9	19	13	1465	1537	1343	1513
rs27020852	112.9521	0.043112	0	0	0	0	1465	1537	1343	1513
rs27020673	112.9953	0.043545	0	0	3	1	1465	1537	1343	1513
rs27004041	113.0388	0.049839	0	1	1	1	1465	1537	1343	1513
rs27003849	113.0886	0.046578	0	0	0	0	1465	1537	1343	1513
rs27034039	113.1352	0.195687	2	0	5	7	1465	1537	1343	1513
rs27004159	113.3309	0.12271	2	0	0	0	1465	1537	1343	1513
rs27017835	113.4536	0.099289	15	18	14	17	1465	1537	1343	1513
rs27007024	113.5529	0.051946	1	0	0	0	1465	1537	1343	1513

rs27006823	113.6049	0.050209	0	0	1	0	1465	1537	1343	1513
rs28293191	113.6551	0.050287	0	0	0	0	1465	1537	1343	1513
rs28278646	113.7053	0.030961	1	1	1	2	1465	1537	1343	1513
rs28278522	113.7363	0.095541	1	2	4	6	1465	1537	1343	1513
rs28240961	113.8318	0.04622	0	1	4	1	1465	1537	1343	1513
rs28240759	113.8781	0.059001	0	0	3	1	1465	1537	1343	1513
rs27043433	113.9371	0.179004	1	4	3	2	1465	1537	1343	1513
rs27013022	114.1161	0.216996	4	1	2	0	1465	1537	1343	1513
rs27000564	114.3331	0.04914	0	0	0	0	1465	1537	1343	1513
rs27000538	114.3822	0.00265	0	0	0	0	1465	1537	1343	1513
rs27000524	114.3849	0.050748	0	1	0	1	1465	1537	1343	1513
rs27044914	114.4356	0.042467	2	1	8	13	1465	1537	1343	1513
rs27029785	114.4781	0.007345	1	0	0	1	1465	1537	1343	1513
rs27029776	114.4854	0.044864	0	0	0	0	1465	1537	1343	1513
rs27029736	114.5303	0.004875	0	0	0	0	1465	1537	1343	1513
rs27029722	114.5352	0.022672	7	11	11	20	1465	1537	1343	1513
rs27029716	114.5578	0.127773	2	6	2	0	1465	1537	1343	1513
rs27029593	114.6856	0.049993	0	0	3	2	1465	1537	1343	1513
rs27015760	114.7356	0.037085	0	1	2	4	1465	1537	1343	1513
rs27015681	114.7727	0.049144	0	0	1	0	1465	1537	1343	1513
rs27015604	114.8218	0.000986	0	0	0	0	1465	1537	1343	1513
rs27015601	114.8228	0.008582	0	0	0	0	1465	1537	1343	1513
rs27015568	114.8314	0.113397	0	0	0	0	1465	1537	1343	1513
rs27015532	114.9448	0.040745	2	3	5	7	1465	1537	1343	1513
rs27015499	114.9855	0.006048	0	0	0	0	1465	1537	1343	1513
rs27015476	114.9916	0.047944	0	0	0	0	1465	1537	1343	1513
rs27000355	115.0395	0.001983	1	0	3	4	1465	1537	1343	1513
rs27000347	115.0415	0.044818	0	0	7	5	1465	1537	1343	1513
rs27000241	115.0863	0.033708	0	0	0	1	1465	1537	1343	1513
rs27000182	115.12	0.056164	0	0	0	0	1465	1537	1343	1513
rs27047920	115.1762	0.098065	4	6	8	6	1465	1537	1343	1513
rs27031548	115.2743	0.060163	0	0	0	0	1465	1537	1343	1513
rs27031489	115.3344	0.001769	0	0	0	0	1465	1537	1343	1513
rs27031483	115.3362	0.290912	3	1	0	2	1465	1537	1343	1513
rs27015935	115.6271	0.045285	0	0	0	0	1465	1537	1343	1513
rs27015865	115.6724	0.041698	3	0	8	5	1465	1537	1343	1513
rs27001667	115.7141	0.013967	0	0	0	1	1465	1537	1343	1513
rs27001625	115.7281	0.200309	1	1	1	2	1465	1537	1343	1513
rs27031005	115.9284	0.100873	0	0	1	0	1465	1537	1343	1513
rs27030879	116.0292	0.098744	1	0	3	1	1465	1537	1343	1513
rs27017443	116.128	0.105351	0	0	0	0	1465	1537	1343	1513
rs27002550	116.2333	0.09068	3	2	1	5	1465	1537	1343	1513
rs27002411	116.324	0.049232	5	4	12	14	1465	1537	1343	1513
rs27002336	116.3732	0.047934	0	2	1	0	1465	1537	1343	1513
rs27046975	116.4212	0.048301	2	0	2	2	1465	1537	1343	1513
rs27029221	116.4695	0.035979	0	0	4	1	1465	1537	1343	1513
rs27029149	116.5055	0.047339	2	5	20	13	1465	1537	1343	1513

rs27029051	116.5528	0.038216	0	0	0	0	1465	1537	1343	1513
rs27029019	116.591	0.049428	0	0	1	0	1465	1537	1343	1513
rs27028977	116.6404	0.047292	2	0	1	10	1465	1537	1343	1513
rs27013254	116.6877	0.111599	0	0	1	1	1465	1537	1343	1513
rs27013222	116.7993	0.050355	0	0	0	1	1465	1537	1343	1513
rs27013120	116.8497	0.047307	5	2	5	8	1465	1537	1343	1513
rs26999779	116.897	0.037274	0	0	0	1	1465	1537	1343	1513
rs26999742	116.9343	0.181626	1	0	0	1	1465	1537	1343	1513
rs27030727	117.1159	0.220998	0	0	1	0	1465	1537	1343	1513
rs27016791	117.3369	0.05324	0	0	0	0	1465	1537	1343	1513
rs27016763	117.3901	0.049166	8	10	22	16	1465	1537	1343	1513
rs27002784	117.4393	0.050031	0	1	0	3	1465	1537	1343	1513
rs27002646	117.4893	0.040992	0	0	0	0	1465	1537	1343	1513
rs28290786	117.5303	0.203755	0	0	0	0	1465	1537	1343	1513
rs28259342	117.7341	0.198074	2	1	1	3	1465	1537	1343	1513
rs28237377	117.9322	0.207576	1	0	0	0	1465	1537	1343	1513
rs27015278	118.1397	0.048177	3	1	8	7	1465	1537	1343	1513
rs27015172	118.1879	0.013966	0	0	2	0	1465	1537	1343	1513
rs26999557	118.2019	0.061059	1	0	0	0	1465	1537	1343	1513
rs26999511	118.2629	0.024426	0	0	0	0	1465	1537	1343	1513
rs26999466	118.2874	0.056443	1	0	1	1	1465	1537	1343	1513
rs26999427	118.3438	0.191128	0	0	3	0	1465	1537	1343	1513
rs27012584	118.5349	0.203375	6	6	8	6	1465	1537	1343	1513
rs27028462	118.7383	0.197724	2	0	0	0	1465	1537	1343	1513
rs27013535	118.936	0.198311	1	0	0	0	1465	1537	1343	1513
rs26999958	119.1343	0.203377	0	2	0	1	1465	1537	1343	1513
rs27015098	119.3377	0.202447	0	0	2	0	1465	1537	1343	1513
rs26998883	119.5402	0.195963	0	0	1	0	1465	1537	1343	1513
rs27029451	119.7361	0.192897	1	3	7	3	1465	1537	1343	1513
rs27013814	119.929	0.182785	1	1	2	1	1465	1537	1343	1513
rs3726373	120.1118	0.22561	0	0	0	5	995	1014	822	931
rs26981081	120.3374	0.398617	0	0	0	2	995	1014	822	931
rs26950944	120.736	0.049313	0	0	0	0	995	1014	822	931
rs26950869	120.7853	0.103238	0	0	3	0	995	1014	822	931
rs26950680	120.8886	0.049231	0	2	5	3	995	1014	822	931
rs27028258	120.9378	0.197693	0	0	1	0	995	1014	822	931
rs6296805	121.1355	0.400708	0	0	3	1	995	1014	822	931
rs27027359	121.5362									
			Total recombinants							
	Average dis	0.127566629	1244	1293	1100	1192				
