# Supplementary material for: Patterns of Recombination Activity on Mouse Chromosome 11 Revealed by High Resolution Mapping
Source: PLoS One. 2010 Dec 8;5(12):e15340. doi: 10.1371/journal.pone.0015340 (PMC2999565; doi:10.1371/journal.pone.0015340)
Supplement: Table S2 — Intervals with sex-specific differences in recombination rates. Female and male numbers of recombinants are presented together with p-values of the difference calculated by Fisher's exact test and q-values correcting for multiple testing (see Material and Methods). All intervals with q<0.1 are included. (PDF) [file pone.0015340.s002.pdf]

Interval Proximal End (Mb)	Interval Size (Mb)	Female Recombinants	Male Recombinants	p_{FET}	q
9.495185	0.180822	12	0	0	0.0246
9.779034	0.133499	23	4	0.0003	0.0243
16.73894	0.046124	45	15	0.0006	0.0124
18.761097	0.059885	21	3	0	0
20.763918	0.013857	5	29	0	0
35.354832	0.049615	6	21	0.0012	0.0459
43.950743	0.083999	13	0	0	0.0080
46.409431	0.055371	8	40	0	0
49.422563	0.037579	10	40	0	0
49.750579	0.055976	22	53	0	0.0080
51.189663	0.060047	5	23	0	0.0080
59.896347	0.049837	4	26	0	0
62.892059	0.003213	3	35	0	0
81.432617	0.05022	15	0	0	0.0062
83.381848	0.049715	2	17	0	0.0099
88.965785	0.056871	32	9	0.0007	0.0213
93.334399	0.050648	20	2	0	0.0099
99.924114	0.042934	13	0	0	0.0099
100.623649	0.049658	5	20	0.0008	0.0246
106.429329	0.065332	2	19	0	0.0080
107.735979	0.051248	8	29	0	0
107.787227	0.050902	24	47	0.0014	0.0373
112.308483	0.042432	42	8	0	0.0196
113.135218	0.195687	1	12	0.0005	0.0243
114.435607	0.042467	3	21	0	0.0099
115.04151	0.044818	0	12	0	0.0080
116.324016	0.049232	9	26	0.0009	0.0366
116.505462	0.047339	7	33	0	0
